# Checking assumptions: advancing the analysis of sex and gender in health sciences

**DOI:** 10.1186/s13293-025-00803-7

**Published:** 2026-01-02

**Authors:** Katherine Tombeau Cost, Eva Unternaehrer, Jens C. Pruessner, Alex Abramovich, Kristin Cleverley, Peter Szatmari, Meng-Chuan Lai

**Affiliations:** 1 Department of Psychology, University of Waterloo, Waterloo, ON Canada; 2 Department of Psychiatry and Behavioural Neurosciences, McMaster University, Hamilton, ON Canada; 3 Child and Adolescent Psychiatric Research Department, University Psychiatric Clinics Basel, University of Basel, Basel, Switzerland; 4 Department of Psychology, University of Konstanz, Constance, Germany; 5 Institute for Mental Health Policy Research, Centre for Addiction and Mental Health, Toronto, ON Canada; 6 Dalla Lana School of Public Health, University of Toronto, Toronto, ON Canada; 7 Campbell Family Mental Health Research Institute, Centre for Addiction and Mental Health, Toronto, ON Canada; 8 Lawrence S. Bloomberg Faculty of Nursing, University of Toronto, Toronto, ON Canada; 9 Department of Psychiatry, Temerty Faculty of Medicine, University of Toronto, Toronto, ON Canada; 10 Centre for Brain and Mental Health and Department of Psychiatry, The Hospital for Sick Children, Toronto, ON Canada; 11 Autism Research Centre, Department of Psychiatry, University of Cambridge, Cambridge, United Kingdom; 12 Department of Psychiatry, National Taiwan University Hospital and College of Medicine , Taipei, Taiwan

**Keywords:** Sex, Gender, Dichotomisation, Validity, Bias, Measurement error, Residual confounding, Statistical power

## Abstract

**Background:**

Sex and gender are dissociable constructs, each including multiple components. Based on the analytic problems associated with dichotomising continuous variables, we aimed to synthesize a new approach to collecting and analysing sex and gender data in health research, in contrast to the conventional use of dichotomous tickboxes to code sex/gender.

**Methods:**

Using a literature review and data simulations, we examined the magnitude of the statistical and methodological problems associated with the use of a single dichotomised sex/gender variable, including construct validity, predictive validity, measurement error, residual confounding, misclassification and bias due to cut points, power, and representative sampling.

**Results:**

Using the dichotomous sex/gender predictor rather than a continuous sex/gender predictor increased residual confounding up to 80% and misclassification of individual participants up to 50%. Further, there was substantial bias in model parameters when continuous sex/gender variables were dichotomised. Finally, we demonstrate that using the dichotomous sex/gender predictor decreased statistical power, in some cases by more than 50%.

**Conclusions:**

Using a dichotomous sex/gender predictor in place of continuous sex/gender predictors, when applicable, has profound impacts on the modelling and the validity of statistical inferences. Accordingly, we proposed measurement and analytic strategies for new multi-variable data collection and analyses of existing binarized data in relation to sex and gender, to reduce these statistical problems and improve model quality.

**Supplementary Information:**

The online version contains supplementary material available at 10.1186/s13293-025-00803-7.

## Abstract (plain language summary)

In health research, sex and gender are often simplified into binary categories like “male” or “female,” but this overlooks their complexity. Sex refers to a collection of biological traits (like hormones and chromosomes), while gender involves identity, roles, power dynamics, and social context. Treating these constructs as exchangeable or with simple either/or options can misrepresent important differences and weaken research findings. This paper shows how using binary sex/gender categories instead of more precise, continuous measures leads to serious problems. Through simulations, we demonstrate that binary measures of sex or gender cause misclassification, reduce statistical power, bias results, and hide meaningful effects—especially when biological or social traits vary widely within groups. These issues can result in incorrect conclusions about health outcomes. We recommend that researchers move beyond binary tickboxes. Instead, we suggest measuring specific components of sex and gender, like hormone levels or gender expression, using validated tools. As is best practice, we also advocate for asking at least two separate questions: one about sex assigned at birth and one about current gender identity, recognising that gender identity may change across the lifespan. Improved measurement, data collection, and analysis methods can uncover hidden patterns and provide more unambiguous, actionable, and inclusive insights. By capturing the real complexity of sex and gender in data collection, researchers can improve the validity, usefulness, and fairness of biomedical and health research.

**Supplementary Information:**

The online version contains supplementary material available at 10.1186/s13293-025-00803-7.

## Highlights

- Sex and gender are distinct and each has multiple components. Sex involves biological traits; gender involves identity, roles, power dynamics, and social context. Labelling participants as just “male” or “female” is oversimplified and can misrepresent complex realities.
- Dichotomising leads to poor science. Using binary sex/gender variables reduces precision, power, and validity in statistical models, with up to 80% of variation lost with binary variables and misclassification affecting up to 50% of cases. In models that include interaction terms or may have small effect sizes, bias and reduced power are especially problematic.
- Better measurement is possible. Using validated tools and continuous or multi-category measures of sex/gender (e.g., hormone levels, gender roles) will support inclusive, precise data to improve discovery and lead to more valid, equitable health research.

**Supplementary Information:**

The online version contains supplementary material available at 10.1186/s13293-025-00803-7.

## Statement of the problem

In biomedical research, sex and gender are frequently treated as equivalent dichotomous variables. They are frequently the “go-to” exemplar of a nominal, binary/dichotomous variable in statistical textbooks. However, sex and gender neither represent a unitary nor a truly binary construct. Many authors have previously emphasized the need to distinguish the constructs of sex versus gender [1–5]. Sex describes the biological attributes of an organism including chromosomal, gonadal, genital, and hormonal sex (Table 1). Historically, the “measurement” of “sex” has been genital sex assigned at birth or prenatal ultrasonic assessment based on the appearance of the infant’s genitals. In contrast to the biological sex components, gender is defined in psychological, sociocultural, and contextual terms (Table 1), including individual characteristics, cultural and historical context, and developmental stages. Further, gender is a social construct involving power structure, historically stemming and evolving from sex differences in physical and reproductive aspects. Thus, gender is a construct that relies on reproductive differences between bodies to generate a structure for social relations and processes [6]. Taken together, to become useful predictors, modifiers, and covariates, sex and gender require valid and unambiguous measurement and analysis.

Here we focus on the problems that arise in data analysis and interpretation with the conventional framework of sex and gender as tautological, binary/dichotomous variables. While our paper focuses primarily on research with human participants, the limitations associated with the use of dichotomised measures of continuous components of sex can also apply to non-human animal research. While there are a variety of expressions in sex physiologically and the way sex is determined is variable across species, the recommendations made here apply to humans and mammals including common model species such as mice, rats, or primates. As an example, hormone levels (e.g., testosterone) may be variable in same sex co-housed conspecifics, introducing unmeasured variance in the analysis through the nominal genital sex variable [7].

In particular, we are concerned with the measurement of sex and gender constructs. While some studies have begun to include continuous markers of sex and gender and have therefore been able to elucidate the biological and socio-cultural mechanisms of health and disease [8], most studies continue to rely on a convenient heuristic of dichotomised sex or gender (and even conflating the two), based on sex assigned at birth, to determine if a sex difference exists or draw conclusions about sex differences (yet with limited elucidation on potential mechanisms) [9]. In humans, while the presence of a Y-chromosome (or more precisely, the SRY gene) in a fertilized egg can set in motion a different series of events than the absence of a Y chromosome, the Y chromosome per se is unlikely the sole *mechanistic* cause of all observed sex and gender differences. Rather, any effect of the Y-chromosome (or a lack thereof) on sex differences as well as downstream gender-related features and experiences is mediated in parallel or serially by multiple biological and sociocultural processes that vary considerably within the group with a Y chromosome and within the group without a Y chromosome. The real effect may come from sex-related or from gender-related processes, both with mechanistic implications that are dimensional in nature, but effects can be lost if dichotomised male/female is used in analysis and subsequent interpretation [9, 10]. Likewise, making inferences based on the nominal classification of the genitals in model species, such as rats, mice, and primates mask effects which can be related to other aspects of physiology that demonstrate substantial within-sex variations [11]. Variable selection and measurement, specifically the use of a dichotomised proxy variable, determine the conclusions that can be drawn from a model [12].

### Statistical problems with dichotomous sex and gender

The meaning of a self-report measure is created by the participant. When participants respond to a dichotomous tickbox, we do not know what meaning (assigned sex at birth, gender identity, or another component) they apply to the question, or what it means when participants do not answer the question. This is an issue of **construct validity** – the degree to which an instrument measures the construct it purports to measure. The primary questions of validity are: [1] what are we *attempting* to measure when we measure sex or gender with a single dichotomous variable? and [2] what are we *actually* measuring when we measure sex and gender with a single dichotomous variable? Average differences between men and women may be interpreted as reflecting biological differences instead of impact from gender and gendered contexts (or vice versa) [13]. Such misinterpretation may stem from the inevitable tangle of sex and gender in human studies [5], but the convenient heuristic introduces bias in the model parameters, affecting analysis and interpretation. The dichotomous tickbox does not disambiguate physiological attributes from social circumscription, neither for the participant attempting to answer the question nor for the researcher attempting to test a hypothesis.

**Predictive validity** is jeopardized when sex and gender are measured as a single dichotomous variable at a single time point. Predictive validity is the degree to which the way we have operationalised sex and gender today (or in the past) is able to accurately predict other measures of sex and gender in the future. This can include gender expression, but also how individuals born with genitals that do not fit normalized definitions of female and male bodies are approached at birth [14, 15]. Likewise, the ways in which sex and gender are defined in different cultural contexts also call into question the predictive validity of dichotomous sex and gender in cross-cultural research. The ways that sex and gender are constructed and defined has changed, and with it the clinical relevance of these definitions [16].

The dichotomisation of *any* continuous variable increases **measurement error**. Measurement error is the difference between the measured and the true value of the construct. Measurement error occurs when the underlying continuous distributions of sex and gender components are masked with a dichotomous variable. When the difference between the means is small in any two-sample comparison, dichotomisation over-estimates the amount of variance between groups while under-estimating the amount of variance within groups [17]. Large **measurement error introduces residual confounding** [18], which is the bias or distortion that remains in the model after confounding variables have been controlled for. The variance not explained by the dichotomous sex/gender predictor, that may have been explained by the continuous sex/gender predictor, remains in the model as residual confounding. This variance is then not actually statistically controlled, contrary to intention and reported methods. Thereby, the dichotomous tickbox methods creates large error variances which create Type I and II errors. Results derived from the dichotomous sex/gender predictor are at risk of being spurious and non-replicable.

Dichotomisation inherently relies on **cut-points**. A cut-point is the value at which continuous data are split into discrete categories. Cut-points can be evidence-based, statistically based, or arbitrary (e.g., driven by policy or economic needs). Any individual near the cut-point will be classified as belonging to one side or the other, quantified as being equally different despite substantial variability [19]. A binary tickbox sex/gender approach has one cut-point. Some variables associated with sex are multi-categorical (i.e., chromosomal sex) and cut-points are true group-wise distinctions. However, some sex and gender variables are continuous (i.e., hormone levels, gender role/expression). While it may seem simple to have multiple cut-points in gender identity (e.g., man, woman, trans man, trans woman, non-binary), the number of cut-points and where exactly to place them poses a huge challenge in any attempt to categorise a continuous variable [19–21]. This may only result in a “gender trinary” that problematically others gender-diverse individuals: men, women, “others” [5]. Cut-points can produce misclassification based on dichotomous labels despite continuous attributes.

Additionally, where one places the **cut-points introduces bias** to the data [22]. If the bias introduced by the cut-point(s) is small, the data will still fit a χ^2^ distribution and can be analysed as binary data. But if the cut-point(s) introduce more bias, the data no longer fit the χ^2^ distribution and inferences will be incorrect [22]. Depending on where one places cut-points in truly uncorrelated data, one can “change a zero correlation to a monotonically increasing function, a decreasing function, or any shape in between” [22, p.51]. Some of the bias will be reflected in the decrease in the standardised estimate of the effect in the model. Multiple cut-points can also introduce multiple testing with pairwise comparisons and inflation of Type I error [23]. Multiple cut-points in continuous variables will vary between studies, cultures, and contexts, which affects predictive validity [23]. References to the previously established cut-point in the literature reifies a binary cut-point of sex and gender without ever examining validity or clinical significance [20].

Together with questionable validity, a high degree of measurement error, and arbitrary cut-points, we also lose statistical **power**. Power is the likelihood to detect the effect of a variable when the effect is present. Statistical power depends on the pre-selected statistical significance of the test (alpha), the magnitude of the effect to be detected, and the sample size. First, a dichotomous sex/gender variable prevents the detection of any non-linear relationships [24]. Second, the method of dichotomisation determines the reduction in power. For example, under a bivariate normal distribution of two continuous variables with ρ_xy_ = 0.4, the reduction in power associated with dichotomisation of sex/gender at the median would be equivalent to discarding 36% of the data [24, 25]. If dichotomisation of sex/gender is at the mean, the further we move away from the mean due to sample-specific characteristics, the larger the loss of power and effective loss of sample size [19]. When the dichotomous sex/gender variable is used, power is reduced and thereby a larger sample is required to detect the effect. The effective loss of sample size due to the reduced power is a function of the method of dichotomisation. When the dichotomisation moves further from the mean, the difference between the phi-coefficient (i.e., the measure of linear association between bivariate-discrete distribution) and the tetrachoric correlation (i.e., the measure of linear association between bivariate-normal distribution) increases [24, 26], thereby increasing the uncertainty that the results are true and robust. Loss of such a large proportion of data due to decreased power is troublesome, given the efforts and costs in data collection, compounded by already small and underpowered studies [19].

A **representative sample** is important when we attempt to make inferences from the results of statistical analyses to the larger population. Creating a tickbox for dichotomous sex/gender perpetuates informational erasure by excluding individuals whose biological status and gender identities are not represented or who regard the categorisation as inappropriate or offensive [27]. The prevalence estimates of non-binary sex and gender components (Table 1) suggest that we are implicitly excluding or failing to account for 2.3–3.3% of the population when taking a dichotomous approach. This prevalence is likely to increase further as there is a significant increase in the number of children and youth expressing gender diversity and/or experiencing gender dysphoria in the last 10 years [28, 29].

**Fig. 1:**
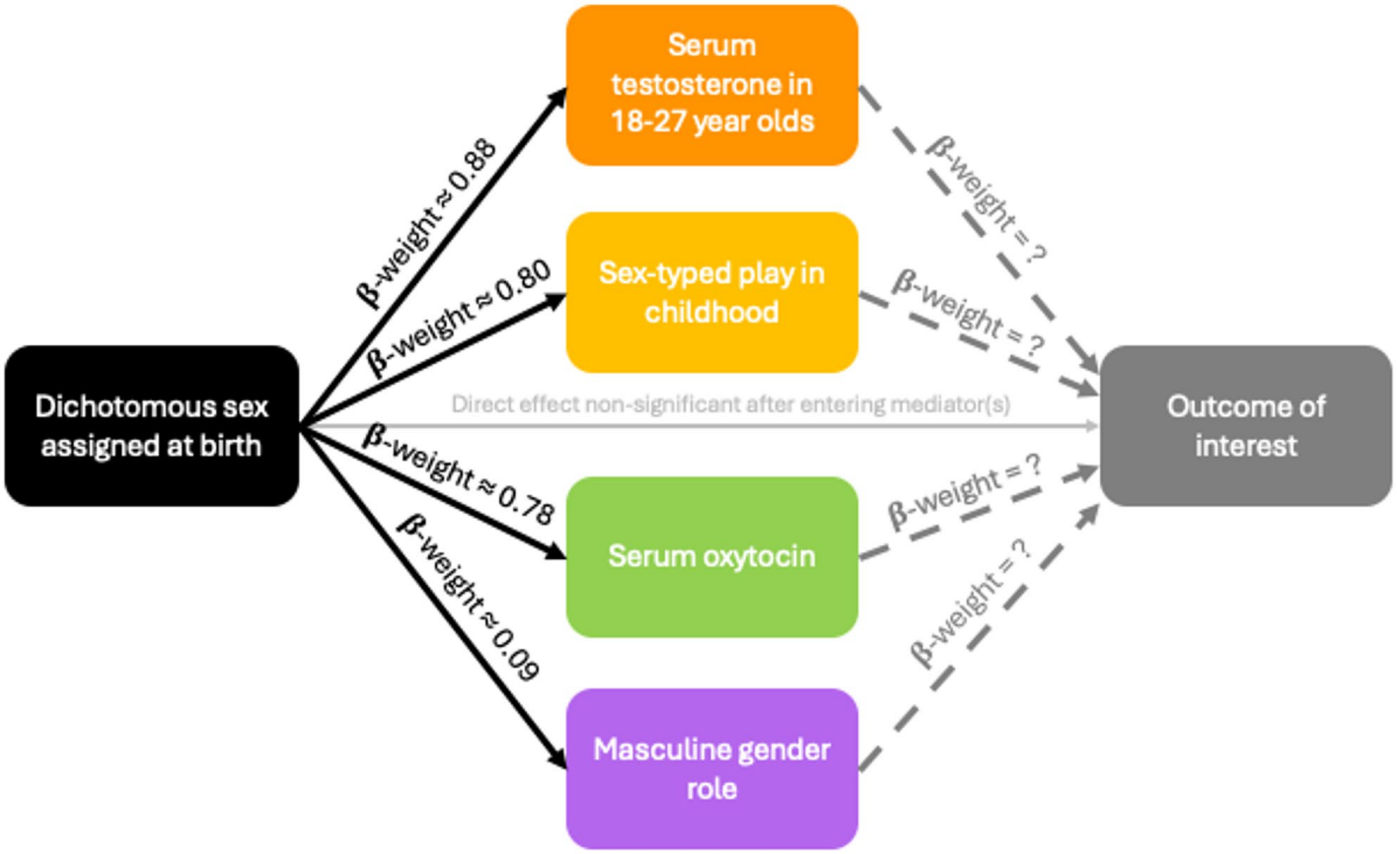
In health science studies, researchers typically collect and analyse a binary distinction based on sex assigned at birth. However, continuous components/dimensions underlying the constructs of sex and of gender, such as gradations in biological manifestations and gender expression/role, are frequently the true drivers of observed patterns. This concept can be illustrated by a mediation model, where sex is assigned at birth (X) and experiences of hormonal milieu or gendered norms (M), for example, manifest prior to the measurement of the outcome of interest (Y). Thus, these unmeasured/hidden dimensions act as mechanistic variables whose influences on the outcome likely completely mediate the observed associations with sex assigned at birth. Mechanistic mediation may be occurring in parallel (as shown in the figure) or serially. While it is unnecessary (and often infeasible) to enter all possible sex-related and gender-related mechanistic variables into analyses, the hypothesised mechanistic driver(s) of a given association can and should be formally tested. β-weight = standardised regression coefficient

Here we used simulation methods to demonstrate the detrimental effects of dichotomisation of the continuous (or quasi-continuous) components (e.g., hormone levels, gender role/expression, polygenic scores related to biological components of sex) of sex and gender on analysis. Continuous components of sex and gender are often the underlying or mechanistic, yet unmeasured, variables of interest in sex and gender research (Fig. 1). First, to illustrate the increase in residual confounding with the dichotomous sex/gender predictor, we computed loss of explained variance (R^2^) in linear regression models when using a dichotomous sex/gender predictor vs. continuous sex/gender predictor. Second, to illustrate the misclassification of individuals by cut-points despite continuous attributes, we computed the amount of overlap between the continuous distributions of sex/gender predictors. Third, to illustrate the statistical bias in model parameters introduced by the dichotomous sex/gender predictor, we calculated the proportional loss of standardised regression coefficient (β-weight) when using the dichotomous sex/gender predictor vs. the continuous sex/gender predictor. Finally, to illustrate the loss of power associated with the dichotomous sex/gender predictor, we computed the difference in power in linear regression models when using the dichotomous sex/gender predictor vs. the continuous sex/gender predictor.

## Methods

### Data simulation

**Fig. 2:**
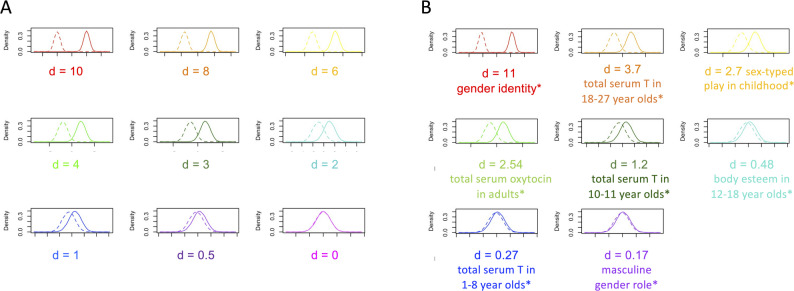
Simulated data distributions. (**A**) Hypothetical simulated continuous variable distributions measured on a theoretical standardised scale rangingfrom 0 (maximal female/woman/feminine prototype) to 10 (maximal male/man/masculine prototype) with varying degrees of differences (Cohen’s d;d = 0–10) between female/woman/feminine (straight line) and male/man/masculine (dashed line). (**B**) Literature-based simulated continuous variabledistributions measured on a standardised scale ranging from 0 (maximal female/woman/feminine prototype) to 10 (maximal male/man/masculine prototype)with varying degrees of differences (Cohen’s ds extracted from published literature; d = 0.17–11.17; see Table 1) between female/woman/feminine(straight line) and male/man/masculine (dashed line). *gender identity [37]; total serum testosterone in 18–27 year-olds [39]; sex-typed play in childhood[37]; total serum oxytocin in adults mean age 34.9 ± 6.2 years [40]; total serum testosterone in 10–11 year-olds [38]; body esteem in 12–18 year-olds [42];total serum testosterone in 1–8 year-olds [38]; masculine gender role [41]

Data were simulated using R (version 4.0.2) [30]. Packages used for plotting and generating statistical parameters included “sm” [31], “ggplot2” [32], “psych” [33], “QuantPsych” [34], “pwr” [35] and “InteractionPoweR” [36]. For the theoretical sex and gender predictor variables, we simulated data for nine theoretical continuously distributed components of sex/gender measured on an arbitrary scale ranging maximal female/woman/feminine prototype to maximal male/man/masculine prototype. For each of the nine theoretical components, we varied the assumed mean difference between the “classical” dichotomised sexes/genders, with values being normally distributed around these different means with a standard deviation of 1. First, we simulated a maximal standardised mean difference (*d* = 10) between female/woman/feminine and male/man/masculine. Next, we simulated data where *d* = 8 and kept decreasing Cohen’s *d* in steps of 1 and 0.5, for a total of nine simulations, until there was no mean difference (*d* = 0). The hypothetical simulated continuous data are illustrated in Fig. 2A. These simulated datasets represent the unmeasured continuous sex and gender components, acknowledging that the true differences between the means will vary as a function of the real component of interest. Additionally, to provide an applied approach to the problem of sex/gender dichotomisation, we also engaged in a literature search to identify components of sex and gender that might be involved in health and disease causal pathways with varying levels of Cohen’s *d*, including gender identity [37]; serum testosterone levels at ages 1–8 years [38], 10–11 years [38], 18–27 years [39]; serum oxytocin levels in adults [40]; sex-typed play in childhood [37]; and masculine gender role [41] (Table 1). To illustrate our point with examples drawn from published literature, we repeated our simulations using these literature-based Cohen’s *d*s (Fig. 2B). Code for all simulations is provided in supplementary materials S1 and S2.

**Table 1:** Components of sex and gender

Components of Sex and Gender	Variable type	Prevalence of non-binary phenotype in humans	Problems with Dichotomising	Examples of Cohen’s d based on assigned sex
Genital sex	continuous	4.6 per 1000 [70]	difficult to classify due to numerous presentations of intersex; problems defining “the norm”, unlikely to be a causal predictor [14, 15]	-
Chromosomal sex	multi-categorical	2.5 per 1000 [71]	unmeasured sex chromosome aneuploidies	-
Gonadal sex	multi-categorical	< 0.05 per 1000 [72]	a lifelong developmental process, which can be stopped or altered even into adulthood [12]. SOX9, FOXL2, ZNRF, NR51A involved in atypical gonadal development and chromosomal-gonadal sex incongruities [73–76]	-
Hormonal sex	continuous	-	Hormone levels: continuously distributed with rhythmic production cycles; Receptor density: continuous across tissues; Receptor functionality: multi-categorical for SNPs; Receptor-hormone interactions: individual genetic differences based on the amount of hormone produced, receptor density, and receptor functionality	*d* = 0.27* Total serum testosterone in 1–8 year-olds [38]; *d* = 2.54** Total serum oxytocin in adults mean age: 34.9 ± 6.2 years [40]; *d* = 3.7** Total serum testosterone in 18–27 year-olds [39]
Gender identity	multi-categorical	6 per 1000 transgender [77]; 10–20 per 1000 gender creative or non-binary gender [78]	ignores individuals who do not identify as “male/boy/man” or “female/girl/woman”; individualistic meaning ascribed to identically named categories; increasing prevalence of gender dysphoria in survey of 18.4 million health records of children and youth age 5–21 from 576 in 2010 to 3,495 in 2014 [28, 29]	*d* = 11.0–13.2.0.2 “core gender identity” based on early androgen exposure [37]
Gender role/expression	continuous	-	a social construct involving power structure, historically stemming and evolving from sex differences in physical and reproductive aspects; not an immutable construct; must include aspects of the individual and the socio-cultural context [1, 5]	*d* = 0.17 Masculine Gender Role [41]; *d* = 0.48 body esteem in 12–18 year-olds [42]; *d* = 2.7 sex-typed play in childhood [37]

*calculated using the midpoint of the given ranges as the mean, assuming the range covers 3 standard deviations of data, thereby calculating the standard deviation for each range as (mean - range_end_point)/3, then calculating pooled standard deviation and Cohen’s d using the derived means and standard deviations

**calculated pooled standard deviation and Cohen’s d from means and standard deviations provided in the reference

### Ethics declaration

Not applicable; data were simulated and drawn from the literature, with no animals or human participants involved in this study, therefore ethics approval was not required for this study.

For each set of analyses, identical samples are drawn to make the model outcomes (R^2^, β-weights, Power) comparable. For our hypothetical outcome variable used in the residual confounding and power analyses, we simulated a normally distributed continuous outcome variable, varying the strength of the association between the simulated sex/gender variables (Fig. 2) and the outcome, from no association (Eq. 1) to a strong positive association (Eq. 2), with the error term being identical across simulations for comparability. For the model comparisons (R^2^, β-weights, Power), we drew random samples (*n* = 100) from the data for each of the nine hypothetical simulated sex/gender datasets (Cohen’s *d* = 0–10). To obtain 95% confidence intervals, we bootstrapped the sample 1000 times. We repeated this procedure for the literature-based simulated data (Cohen’s *d* = 0.17–11.17).

The first two equations were thus defined as follows:

1 $$\mathrm{Outcome} = 0 + 0^\star \text{continuous sex}/ \text{gender + error}$$

2 $$\mathrm{Outcome} = 0 + 2^\star \text{continuous sex}/ \text{gender + error}$$

### Residual confounding

We estimated residual confounding using change in variance explained (*R*^*2*^) between the dichotomous sex/gender predictor linear regression model and the continuous sex/gender predictor linear regression model. We computed the difference in the explained variance in the outcome between the two models (*ΔR*^*2*^, Eq. 3) across all bootstrapping samples, all levels of association with the outcome variable, and all levels of *d* for both hypothetical simulated data (*d* = 0–10) and literature-based simulated data (*d* = 0.17–11.17). More negative values indicate greater reduction in explained variance under the dichotomous sex/gender predictor, hence higher residual confounding. Loss in explained variance ranges from − 1 (complete loss of variance in *y* explained by *x*) to 0 (no loss in variance in *y* explained by *x*).

3 $$\Delta\mathrm{R}^2 = \mathrm{R}^2_{dichotmous} - \mathrm{R}^2_{continuous}$$

### Misclassification

Misclassification was estimated as the percentage of overlap between the continuous distributions for each of the simulated datasets (*d* = 0–10). Misclassification ranges from 0% (no misclassification) to 50% (classification of participants no better than chance), with greater misclassification indicating greater consequence of cut-points.

### Bias in model parameters

Bias in model parameters under the dichotomous sex/gender predictor model were estimated with the proportional decrease in the standardised regression coefficients (*β-weights*). We computed a difference between β-weight_dichotomous_ and β-weight_continuous_ to obtain the change in β-weight between models (*Δβ-weight*; Eq. 4*).* We then calculated the proportional loss in β-weight (*pΔβ-weight*; Eq. 5). Here, proportional loss of standardised regression coefficient ranges from 0% to 100%, with larger values indicating a greater proportional decrease in β-weight under the dichotomous sex/gender predictor, thereby greater bias in the model results.

4 $$\Delta\beta\mathrm{-weight} = \beta\mathrm{-weight}_\mathrm{continuous}-\beta\mathrm{-weight}_\mathrm{dichotmous}$$

5 $$\mathrm{p}\Delta\beta\mathrm{-weight} = \frac{\Delta\beta\mathrm{-weight}}{\beta\mathrm{-weight}_\mathrm{continuous}}$$

### Power

To estimate the loss in power under the dichotomous sex/gender predictor model, we calculated the power to detect a true association between the sex/gender predictor and the outcome, with α = 0.05. Power for the dichotomous sex/gender predictor model (*(1-β)*_*dichotomous*_) was derived using estimates from the dichotomous model. Power for the continuous sex/gender predictor model (*(1-β)*_*continuous*_) was derived using estimates from the continuous model. We then calculated the loss in power (*Δ(1-β)*, Eq. 6) [5].

6 $$\Delta(1-\beta) = (1-\beta)_\mathrm{dichotmous}-(1-\beta)_\mathrm{continuous}$$

To estimate the loss in power and consequent required increase in sample size when a dichotomized sex/gender predictor was included in the model as a primary predictor and as part of an interaction term, we calculated the required sample size to detect a true association between the sex/gender interaction term and the outcome as a function of small to medium correlations (*r* = 0.10; *r* = 0.24) between predictors and outcomes (r_sex/gender, y_, r_x2,y_, r_(sex/gender*x2),y_, r_x1,x2_), with α = 0.05 and power = 0.8 using the “InteractionPoweR” package [36].

## Results

### Residual confounding

**Fig. 3:**
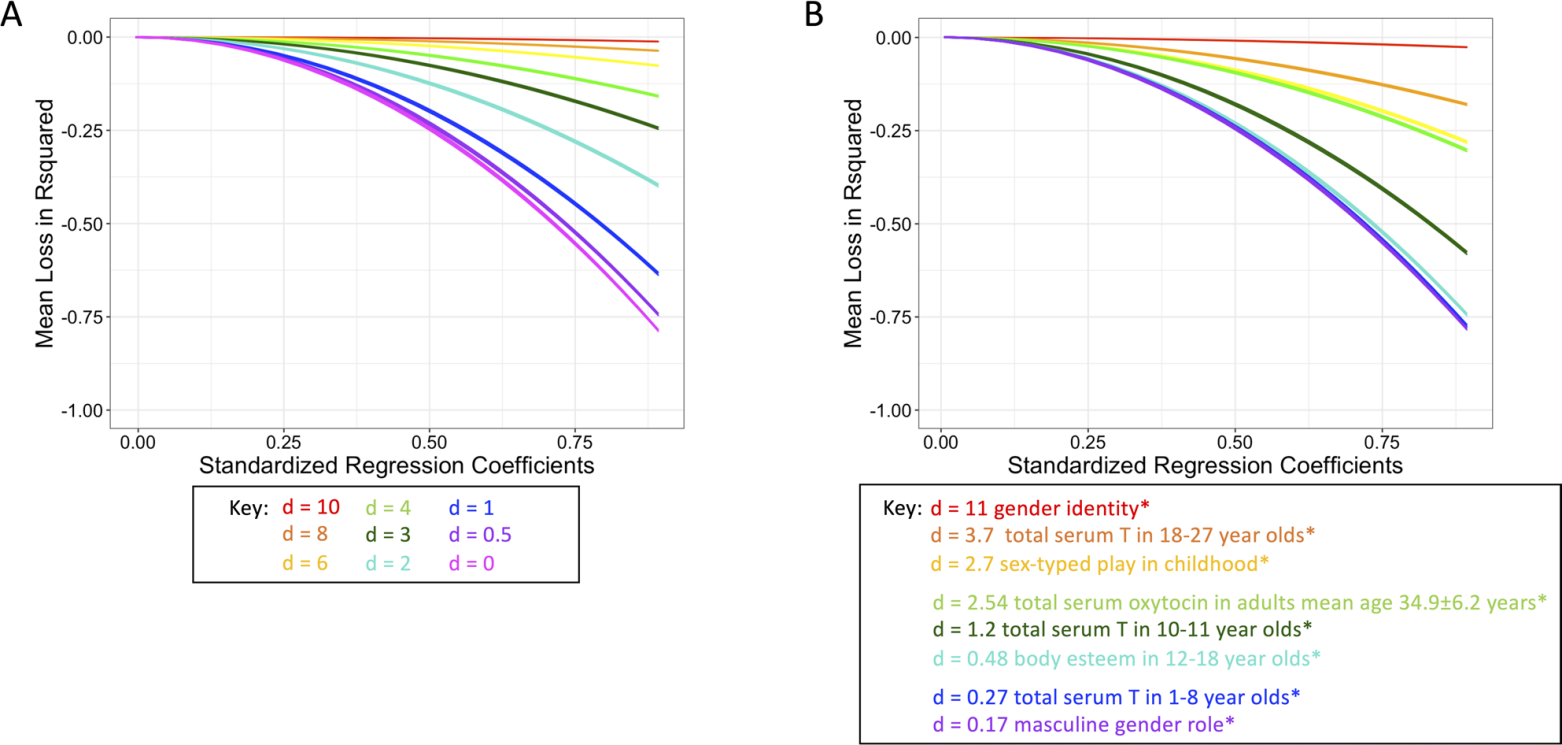
Loss in variance explained. Loss in variance explained (*ΔR*^*2*^*)* by the model as a function of the strength in the underlying association (*β-weight*), under the dichotomous sex/gender predictor. (**A**) hypothetical simulated data (Fig. 2A for the example distributions for each difference). (**B**) literature-based simulated data (Fig. 2B for the example distributions for each difference). *gender identity [37]; total serum testosterone in 18–27 year-olds [39]; sex-typed play in childhood [37]; total serum oxytocin in adults mean age 34.9 ± 6.2 years [40]; total serum testosterone in 10–11 year-olds [38]; body esteem in 12–18 year-olds [42]; total serum testosterone in 1–8 year-olds [38]; masculine gender role [41]

In the hypothetical simulated data, using the dichotomous sex/gender predictor increased residual confounding (*ΔR*^*2*^) as a function of greater regression coefficients (*β-weights)* and smaller standardised mean differences (*d*; Fig. 3 A). When the differences between the means were smaller (*d* < 4) and the standardised regression coefficients were larger (*β-weight* > 0.5), dichotomisation left up to 80% of the variance accounted for by the continuous sex/gender predictor in the model as residual confounding using the dichotomous sex/gender predictor, contributing to large error variance and potentially Type I and II errors.

In the literature-based simulated data, using the dichotomised proxy (male/female) for predictors with low-range *d*’s, such as masculine gender role (*d* = 0.17; [41]), total serum testosterone in children ages 1–8 years (*d* = 0.27; [38]), body esteem in 12–18 year-olds (*d* = 0.48; [42]), or even total serum testosterone in children ages 10–11 years (*d* = 1.2; [38]), was associated with increased residual confounding (*ΔR*^*2*^), particularly for large effect sizes. For mid-range d’s (plasma oxytocin in middle adulthood, *d* = 2.54 [40]; sex-typed play in childhood, *d* = 2.7 [37]; total serum testosterone in 18–27 years, *d* = 3.7 [39]), using the dichotomised proxy for the continuous sex/gender predictors was associated with a 20–30% increase in residual confounding. The only case where residual confounding increased only minimally as a result of using a dichotomised proxy was with core gender identity (*d* = 11 [37]; Fig. 3B).

### Misclassification

**Fig. 4:**
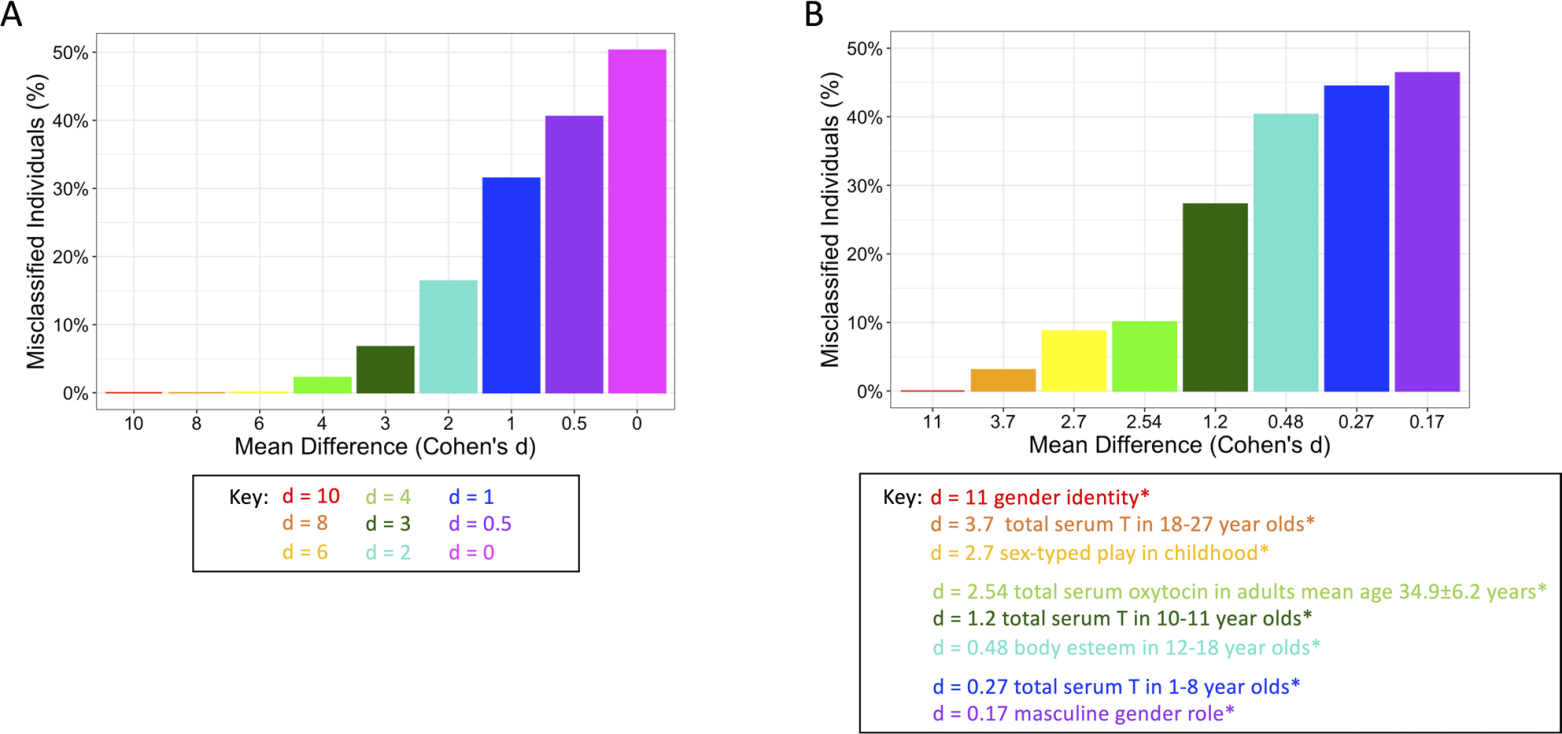
Percent misclassified individuals. Percent misclassification of individuals under the dichotomous sex/gender predictor in place of the continuous sex/gender predictor. (**A**) hypothetical simulated data (Fig. 2A for the example distributions for each difference) (**B**) literature-based simulated data (Fig. 2B for the example distributions for each difference). *gender identity [37]; total serum testosterone in 18–27 year-olds [39]; sex-typed play in childhood [37]; total serum oxytocin in adults mean age 34.9 ± 6.2 years [40]; total serum testosterone in 10–11 year-olds [38]; body esteem in 12–18 year-olds [42]; total serum testosterone in 1–8 year-olds [38]; masculine gender role [41]

In the hypothetical simulated data, although there was no misclassification if the two distributions were exclusive (*d* > 8), more overlap between the distributions (*d* < 4) resulted in an increased percentage of misclassifications based on the dichotomous label, up to 50%, indicating that the dichotomised variable was no better at classifying individuals than flipping a coin (*d* = 0; Fig. 4 A).

In the literature-based simulated data, 25–48% of individuals would have been misclassified when using the dichotomised proxy (male/female) predictors instead of the continuous predictor with low-range *d*’s (≤ 1.2), such as masculine gender role or serum testosterone in children 10–11 years [38]. In the mid-range d’s (2.5–4.0.5.0) such as serum testosterone in 18–27 years [39] or composite of sex-typed play in childhood [37], misclassification due to binary proxy ranged from 6 to 10% (Fig. 4B).

### Bias in model parameters

**Fig. 5:**
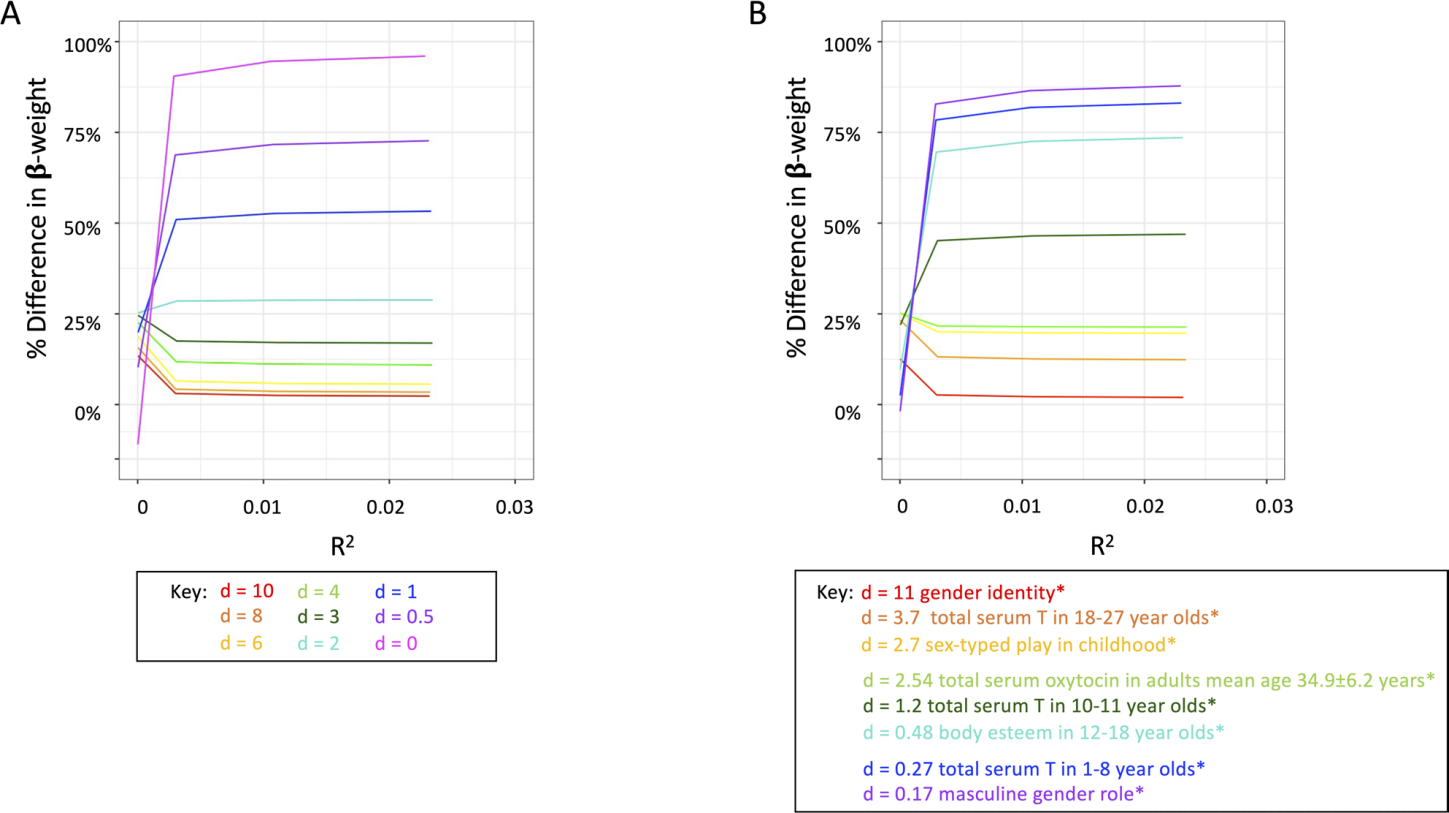
Bias in parameter estimates (proportional decrease in standardised correlation coefficients). Proportional decrease in standardised correlation coefficient (*pΔβ-weight*) of the sex/gender predictor as a function of the proportion of variance explained by the continuous sex/gender predictor (*R*^*2*^_*continuous*_) and the standardised difference between the means (*d*), depending on whether the dichotomous sex/gender predictor is used in place of the continuous sex/gender predictor. (**A**) hypothetical simulated data (Fig. 2A for the example distributions for each difference). (**B**) literature-based simulated data (Fig. 2B for the example distributions for each difference). *gender identity [37]; total serum testosterone in 18–27 year-olds [39]; sex-typed play in childhood [37]; total serum oxytocin in adults mean age 34.9 ± 6.2 years [40]; total serum testosterone in 10–11 year-olds [38]; body esteem in 12–18 year-olds [42]; total serum testosterone in 1-8year-olds [38]; masculine gender role [41]

In the hypothetical simulated data, the bias in model parameters using the dichotomous sex/gender predictor measured with proportional decrease in the standardised regression coefficient (*pΔβ-weight*) ranged 1–10% when the standardised difference between the means was larger (d > 4). When the standardised difference between the means was relatively small (d ≤ 1), proportional bias in model results exceeded 50% (up to 100%), asymptotic at any R^2^ > 0.03, indicating that bias in model parameters was primarily a function of the standardised mean differences (*d*) alone (Fig. 5 A).

In the literature-based simulated data, using the dichotomised proxy (male/female) for predictors with *d* < 1 (i.e., masculine gender role [41], total serum testosterone in 1–8 year-olds [38], body esteem in 12–18 year-olds [42]) resulted in approximately 75% decrease in standardized regression coefficients, while in the case of serum testosterone in 10–11 year-olds (*d* = 1.2) [38] standardized regression coefficients decreased 50% under the dichotomised predictor. When the standardized difference between the means was larger (*d* >2), as with serum oxytocin in adults mean age 34.9 ± 6.2 years (*d* = 2.54) [40], sex-typed play in childhood (*d* = 2.7) [37], and serum testosterone in 18–27 year-olds (*d* = 3.7) [39], standardised regression coefficients were decreased by 12–20%. Standardised regression coefficients were minimally impacted (< 5% decreased) when Cohen’s *d* was very large, as is the case of early hormone environment on gender identity (*d* = 11) [37] (Fig. 5B).

### Loss in power

**Fig. 6:**
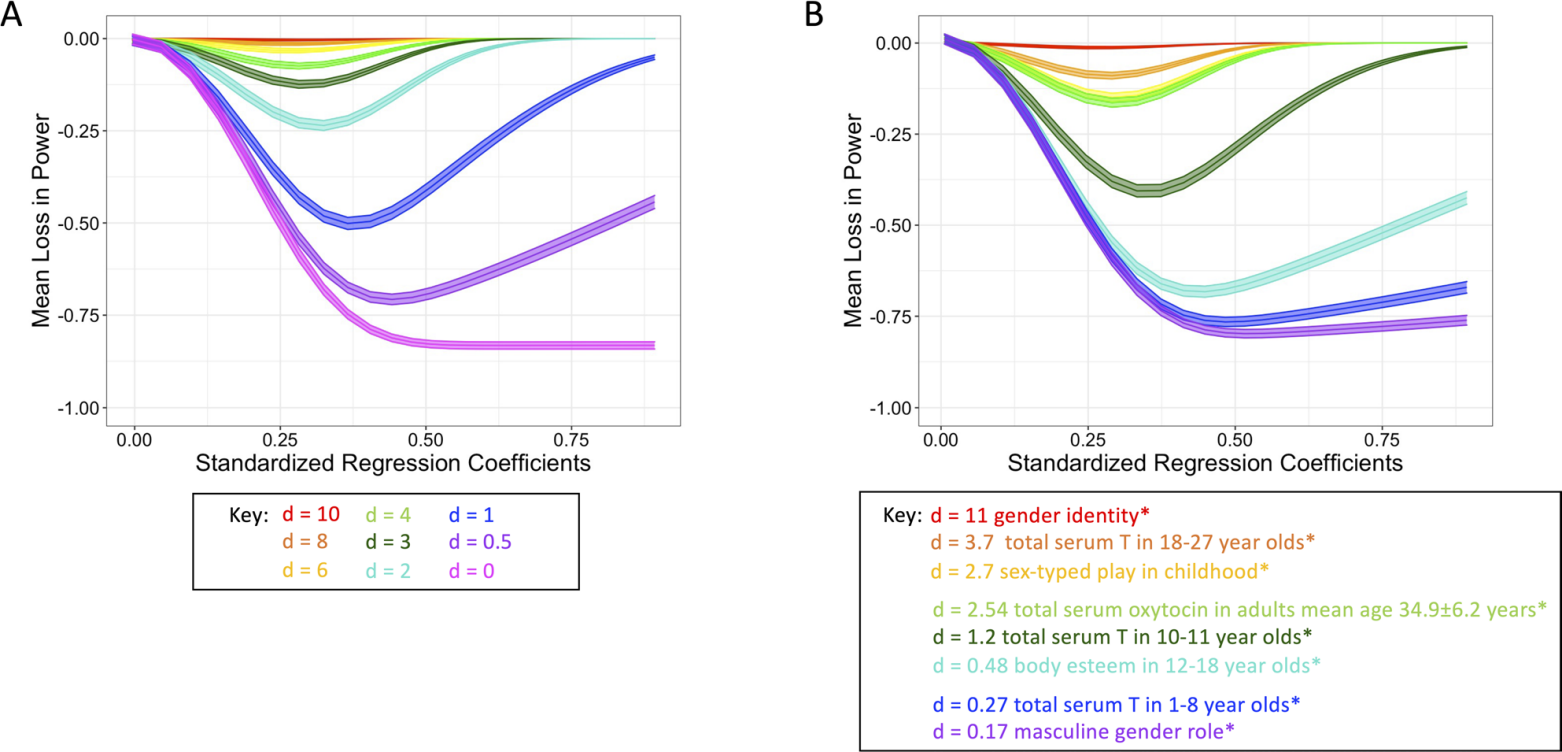
Loss in power. Loss of power of the model as a function of the strength in the underlying association (*β-weight*), when using the dichotomous sex/gender predictor in place of the continuous sex/gender predictor. (**A**) hypothetical simulated data (Fig. 2A for the example distributions for each difference). (**B**) literature-based simulated data (Fig. 2B for the example distributions for each difference). *gender identity [37]; total serum testosterone in 18–27 year-olds [39]; sex-typed play in childhood [37]; total serum oxytocin in adults mean age 34.9 ± 6.2 years [40]; total serum testosterone in 10-11year-olds [38]; body esteem in 12–18 year-olds [42]; total serum testosterone in 1-8year-olds [38]; masculine gender role [41]

In the hypothetical simulated data, when the distributions completely overlapped (*d* = 0), the loss of power (*Δ(1-β)*) exceeded 50% under the dichotomous sex/gender predictor, particularly as standardised regression coefficients (*β-weight* > 0.25) increased (Fig. 6). As the difference between means increased (*d* > 4), the use of the continuous sex/gender predictor was particularly important at small-to-medium standardised regression coefficients (*β-weight* < 0.5). Only when the associations reached large effect sizes (*β-weight* > 0.75) *and* there were large standardized differences between the means (*d* > 4), power became comparable when using either the dichotomous sex/gender predictor or the continuous sex/gender (Fig. 6 A).

In the literature-based simulated data, using the dichotomised proxy (male/female) for predictors with low-range d’s (*d* ≤ 1.2 (masculine gender role [41], total serum testosterone in 1–8 year-olds [38], body esteem in 12–18 year-olds [42], serum testosterone in 10–11 year-olds [38]), resulted in 30–80% loss in power, even when *β-weight* >0.25. Only when *d* >1.2, such as serum oxytocin in adults mean age 34.9 ± 6.2 years (*d* = 2.54) [40], sex-typed play in childhood (*d* = 2.7) [37], and serum testosterone in 18–27 year-olds (*d* = 3.7) [39], and when the association with the outcome variable was very large (*β-weight* >0.63), power was not impacted by dichotomisation (Fig. 6B).

When the dichotomised sex/gender variable was used as a primary predictor and in an interaction term in sample size determination with α = 0.05 and power = 0.8, sample size requirements increased 20.6–40.0%, depending on the constellation of correlations between predictors and outcomes (r_sex/gender, y_, r_x2,y_, r_(sex/gender*x2),y_, r_sex/gender, x2_) in the data (Table 2).

**Table 2:** Loss in power and consequent required increase in sample size when a dichotomised sex/gender predictor was included in the model as a primary predictor and as part of an interaction term. Here we vary two effect sizes, representing small (r = 0.10) and medium (r = 0.24) effects, for main effect of sex/gender on the outcome (r_sex/gender, y_), main effect of another predictor on the outcome (r_x2,y_), their interaction effect on the outcome (r_(sex/gender*x2),y_), and the correlation between predictors (r_sex/gender, x2_). Parameters to calculate sample size were defined as α = 0.05, power = 0.8

*r*_sex/gender, y_	*r*_x2,y_	*r*_(sex/gender*x2),y_	*r*_sex/gender, x2_	N required for power = 0.8 with dichotomous sex/gender predictor	N required for power = 0.8 with continuous sex/gender predictor	% increase in required sample size under the dichotomous model
0.10	0.10	0.10	0.10	1249	769	38.5%
0.10	0.24	0.10	0.10	1137	729	35.9%
0.10	0.10	0.10	0.24	1236	772	37.5%
0.10	0.24	0.10	0.24	1165	752	35.4%
0.24	0.10	0.10	0.10	1231	739	40.0%
0.24	0.24	0.10	0.10	1113	734	34.0%
0.24	0.10	0.10	0.24	1187	725	38.9%
0.24	0.24	0.10	0.24	1157	714	38.3%
0.10	0.10	0.24	0.10	218	173	20.6%
0.10	0.24	0.24	0.10	245	164	33.2%
0.10	0.10	0.24	0.24	250	186	25.6%
0.10	0.24	0.24	0.24	250	161	35.7%
0.24	0.10	0.24	0.10	243	157	35.5%
0.24	0.24	0.24	0.10	240	147	38.8%
0.24	0.10	0.24	0.24	248	169	31.9%
0.24	0.24	0.24	0.24	220	140	36.2%

Effect sizes (small = 0.10, medium = 0.24) are based on Cohen's d converted to r [88], which assumes equal sample sizes. For full code to determine sample size given set power and correlation coefficients, see Supplement 3

## Discussion

Using simulation methods with both theoretical and literature-based standardised differences between the means to demonstrate the impact of dichotomising continuous sex and gender component variables on statistical models, we found that using dichotomous sex/gender variables increased residual confounding; in some cases, up to 80% residual confounding remained in the model. We found that as the standardised difference between the means decreased, the percentage of misclassified individuals increased up to 50% misclassification. We found substantial bias in model parameters when continuous sex/gender variables were dichotomised, particularly when the standardised difference between the means decreased, with up to 100% proportional bias in model parameters. Finally, we found that using dichotomous sex/gender variables decreased power, particularly when the standardised difference between the means was larger and the standardised regression coefficient was smaller. Further, use of a dichotomized sex/gender predictor in an interaction term was associated with decreased power and therefore significantly larger sample sizes would be required (20.6–40% larger) to achieve equivalent power than would be observed with a continuous sex/gender predictor in an interaction term. Based on our review of the literature, variables in the causal pathway that are most likely to create statistical problems include gender role/expression, certain hormone levels in young children (e.g., testosterone), body esteem in adolescents, and certain hormone levels in adults (e.g., oxytocin). However, this list of causal variables with small Cohen’s *d* is certainly not exhaustive and researchers should thoroughly investigate the magnitude of mean differences in mechanistic sex and gender variables a priori, tailored to their specific research questions. In sum, using a dichotomised sex/gender variable in place of continuous sex/gender variables has profound impacts on the statistical model and the validity of the statistical inferences. While we discuss the statistical problems resulting from dichotomisation individually, these problems are occurring simultaneously, synergistically impacting results and subsequent interpretation.

To improve **construct validity**, we suggest researchers measure the true sex/gender variable(s) of interest in the causal chain. Because the dichotomous tickbox carries multiple interpretations, we suggest including a two-step question assessing sex assigned at birth and current gender identity, which has shown high sensitivity and specificity [43–45]. Construct validity might also be improved when “chromosomal sex” is used in place of “genetic sex”, as multiple genes are involved in gonadal and hormonal sex. Sex assigned at birth, chromosomal sex, and gender identity are all multi-categorical variables. However, data analysis can still include all participants, even for categories with low frequency responses as outlined in Table 3. Research based on sex assigned at birth or gender identity should acknowledge the limitations of this approach in causal inferences.

**Table 3:** Measurement and analytic approaches for components of sex and gender

Components of Sex and Gender	Potential Measurement Approaches	Potential Analytic Approaches
Chromosomal sex	karyotype (i.e., XX, XY, XXY, XYY, etc.)	• stratification for imbalanced groups using the appropriate analysis depending on outcome variable type and distribution • non-parametric approaches, i.e., o Kruskal Wallis H test for analysis of variance with small or unequal group sizes o Mann Whitney U test for small or unequal group sizes o Kolmogorov-Smirnov test to compare a small group’s probability distribution to reference group’s or theoretical probability distribution
Gonadal sex	• gene expression in gonadal tissue (i.e., mRNA) • cell type ratios in biopsy samples	• all continuous predictors, therefore logistic or linear regression methods or other methods depending on outcome variable type and distribution
Hormonal sex	• assay from hair, saliva, urine, or blood [39, 79, 80] • hormones with oscillatory fluctuations, require repeated measurement of hormones or covariation of cycle stage in the analysis • genotyping for receptor functionality and receptor-ligand interactions to generate biologically-informed polygenic scores [81, 82] • epigenetics • gene expression (i.e., mRNA) • “deep hormonal characterization” to disentangle interactions between hormonal sex, the receptor genetics, gonadal differentiation and maintenance [73, 81, 83] • hormonal levels do not always align with an individual’s gender identity, depending on medically transitioning or hormone replacement therapy	• all continuous predictors, therefore logistic or linear regression methods or other methods depending on outcome variable type and distribution
Gender identity	• identified as separate from sex assigned at birth and legal sex designation in a 2-step or 3-step question [84] • self-report with multi-categorical gender identities	• stratification for imbalanced groups using the appropriate analysis depending on outcome variable type and distribution • non-parametric approaches, i.e., o Kruskal Wallace H test for analysis of variance with small or unequal group sizes o Kolmogorov-Smirnov test to compare a small group’s probability distribution to reference group’s or theoretical probability distribution o Fisher’s exact test for categorical outcomes with small group sizes with Freeman-Halton extension for tables larger than 2 × 2
Gender role/expression	• self-report questionnaire(s) including gender ideology, gendered behaviour, gender socialization, gender role stress, structural components of gender (feminist identity, old-fashioned sexism, modern sexism, ambivalent sexism, inventory of sexist events) [48, 85] • United Nations Gender Inequality Index as an indicator of cultural/contextual gender norms [86] • difference scores between standardised measures of gender at the individual level and measures of gender norms at the cultural level • interaction terms between individual gender and cultural gender norms could provide a goodness-of-fit or “mismatch” measure, where either conformity or resistance to gender norms may underlie susceptibility to health challenges • interaction terms to identify health outcomes due to discrimination, stigma, and minority stress that arise from cultural expectations of a gender • health outcomes as a product of individual by culture interactions [87]	• all continuous predictors, therefore logistic or linear regression methods or other methods depending on outcome variable type and distribution

The measurement and analytic approaches are not exhaustive, but represent the most likely causal sex and gender components and the most common analytic approaches. See Supplement for resources on non-parametric tests

To enhance **predictive validity** in gender variables, we suggest including measures of gender identity and roles/expressions as contextual and individual-context interactive factors. In the field of economics, contextual factors are included, with data often being adjusted for seasonal fluctuations in consumer behaviour [46]. To apply this concept to the assessment of an individuals’ gender identity and roles/expressions one could take into account the context, such as cultural norms and expectations (Table 3). By including both the personal experience of gender and the context-dependent expectations of gender, we can understand when personal, contextual, or greater variance in the components of sex and gender are associated with health outcomes [47].

To reduce **measurement error and residual confounding**, we suggest hypothesis-driven and evidence-based selection of the particular sex/gender predictor(s) of interest, measured with reliable and valid instruments. At present, measurement error and residual confounding are high because of using an inappropriate dichotomous proxy to measure the true variable(s) of interest.

For components of gender, Smiler and Epstein [48] offer a primer on measurement options (Table 3). Although there are methodological issues related to certain measures of gender, notably older measures of gendered traits [48], relevant measures of gender are available and being developed. While some questionnaires problematically have different versions for male and female participants [49], and others focus only on the hierarchy of gender binary without acknowledging the hierarchy of gender plurality, these dimensional questionnaires still measure gender components better than a binary tickbox. Future editions of these questionnaires should be updated with gender-inclusive versions and language in consultation with gender-diverse individuals. For concerns about participant burden, Smiler & Epstein surveyed 30 questionnaires on components of gender, which ranged in length from five to 240 items, with a mean of 49 items [48]. Nine questionnaires had 20 or less items and only one had more than 100 items. However, if gender components are not adequately captured, a greater burden is put on participants to give their time for research that fails to address critical questions on health outcomes relevant to sex and gender.

Resolving the problem of **cut-points**, which causes **misclassification** and **bias in model results**, is as simple as using a continuous scale when measuring a continuous sex/gender variable and a multi-categorical scale when measuring a multi-categorical sex/gender variable.

While validity, measurement error, and cut-points can only be resolved through changing practices moving forward, **power** can be improved statistically with data that have been or are being collected. First, the reduction in power under a dichotomous sex/gender predictor decreases effective sample size for both models with a single predictor [24, 25] and models with interaction terms (Table 2). For ongoing studies with dichotomous measures, sample size should increase to detect the effect of the continuous sex/gender component, although simply increasing sample size will only address issues around power and not those pertaining to bias in model coefficients or effect size. Second, when running correlation analyses with continuous sex/gender components assessed with a dichotomised measure, the tetrachoric correlation and the biserial correlation, both assuming an underlying continuous distribution, should be used in place of the point-biserial correlation or chi-squared, both assuming a true dichotomous variable [24]. Third, Wainer & Gessaroli [22] suggest a more statistically intensive approach of simulation to recreate the continuous data using the binned data and matching means and variances. Procedures for this method are available in SAS, Stata, and R [50, 51]. Finally, the same approach that addresses the problems of validity, measurement error, and cut-points [17, 19, 21–25, 48, 52] also addresses power: i.e., using continuous measures to assess continuous constructs.

Regarding **representative sampling**, studies that rely on a single dichotomous sex/gender variable should acknowledge the statistical limitations as well as the implicit exclusion associated with this approach. Further, if participants have been excluded from analysis explicitly due to responses on questions on sex assigned at birth and/or gender identity, the reasons for this exclusion and the limitations to generalisation should be clearly acknowledged and justified. When possible, analysis including data on all participants can reduce sampling and reporting bias and facilitate future meta-analysis on a full range of data. While the representativeness of a sample may not impact the types of analyses used, it impacts the way in which the *results* of the analyses can be interpreted and used. To approximate representative sampling, the sample must include a diverse spectrum of sex and gender.

### Improving practice to enhance discovery and theoretical development

When acknowledging the multiple components with multi-categorical and continuous aspects in sex and gender, one can better capture specific and actionable effects on outcomes of interest. Whether these components are associated with biological, contextual, or psychological underpinnings, or the degree of agreement between biology, context, and psychology on health outcomes, can be explored using **factor analyses and latent profile analysis.**

Few studies have collected comprehensive sex and gender information from a representative sample. When available, such data can be examined with exploratory factor analysis to reveal the associations between variables and thereby underlying latent constructs. Factor analytic methods can also be used to isolate components of gender that vary by culture or context. For example, several measures of gender components have inconsistent factor structures across studies, which is likely the product of changing definitions of gender across historical and cultural contexts. Factor structures reflect the underlying multivariate association of the individual questions in representing a latent construct. Gender, a collection of latent construct variables, may be better represented within different contexts by different factor structures, rather than adherence to a set factor structure which may not be persistently valid. Inconsistent factor structures due to measurement non-invariance may actually permit an opportunity to study components of gender that are stable (invariant), and the relevance of non-invariant components of gender in health outcomes cross-culturally and across time [53, 54]. Such comprehensive measurement of multiple aspects of sex and gender will be the foundation to better understand the structure of the components of sex and gender, and the discovery will significantly inform theoretical development of sex and gender effects on health as well as improving best practices in data analysis.

Latent profile analysis is a statistical method to identify hidden or unobserved groups of individuals in a dataset using observed continuous measures. It can obtain the distributions in unobserved groups as well as generating the probability that any individual belongs to any group [55]. In the presence of comprehensive sex and gender measurement, latent profile analysis can estimate how different sex and gender groups are present in a representative sample. This discovery may bring our understanding of sex and gender further using a data-driven, hypothesis-free approach, and to provide statistically supported reduction of sex and gender components.

Some studies across a range of health conditions, including multimorbidity, cardiovascular health, cancer, psychiatry, neurology, and neurodevelopmental conditions have already begun to use some of the methods described above. A gendered focus is important to elucidate socio-cultural mechanisms of gender differences in health outcomes. When evaluating multimorbidity in transgender people, evidence of the gender hierarchy in social determinants of health was evident, with transgender individuals more likely to live in lower income neighbourhoods compared to cis-gender peers [56, 57]. Moreover, transgender individuals had higher rates of multimorbidity and service use than their cis-gender peers, particularly for self-harm and mental health concerns [56]. Another study that included both sex assigned at birth and a composite “gender variable” demonstrated that increased risk factors for cardiovascular events were associated with higher feminine gender composite scores, but not with assigned female sex at birth, when both birth-assigned sex and composite gender score were included in the model [58]. Similarly, in an example from cancer research, a study that evaluated sex assigned at birth as well as gender role variables reported that apparent sex differences in cancer rates were no longer statistically significant after gender role variables were included in the model [59]. In studies examining cancer risk, multiple variables underpinning biological sex as well as sociocultural gender are implicated in the causal pathways to cancer aetiology, treatment effects, and survival, from complement of sex chromosomes (including XX, XY, XXY, and XO genotypes), to the impacts of testosterone and sexual differentiation, to gender norms that prevented the inclusion of women in clinical trials of cancer treatments [60, 61]. The value and importance of distinguishing sex-related versus gender-related constructs to clarify developmental mechanisms, clinical characterisations, as well as intervention and support directions have been well highlighted for neurological, psychiatric, and neurodevelopmental conditions [62–65]. These studies illustrate how our key findings can be applied across a range of disciplines and outcomes of interest in health sciences to improve our understanding of health and diseases.

### Strengths & limitations

We integrated the current state of statistical sciences with sex and gender sciences, conducted robust simulations based on both theoretical and literature-based examples, demonstrated the impact on power and effective sample size when dichotomous sex and gender variables are used in interaction terms, summarised potential measurement and analytic approaches for different sex/gender components, and formulated practical recommendations for researchers. The current study also has several important limitations. First, our survey of the sex and gender literature for examples of mechanistic sex and gender variables is not exhaustive and different research questions will involve different sex and gender variables with different standardised differences between the means that may not be represented in this paper. Second, our table describing common measurement and analysis approaches is not exhaustive. Third, our recommendations are based on our current understanding of the nature of sex and gender variables, but sex and gender science as well as statistical science are dynamic, requiring measurement and analytic approaches be updated as the knowledge base grows.

## Conclusion

There will be the argument that dichotomous sex and gender is how we have always done things, that sex and gender as a dichotomous variable simplifies analysis and interpretation of results, that it is difficult to measure sex and gender with longer questionnaires or bio-assays, that non-binary sex and gender variants are too rare to include, that modern measures of sex and gender will increase the cost of projects and thwart the progress of science. Such arguments are not new and have been made in the past about ethics oversight and protection in research with animals and human participants, yet we all acknowledge the righteousness of this advance and how it has improved the validity of the conclusions we draw from research, despite bureaucratic challenges [66]. Just noting the importance of including non-males in research met with familiar pushback: that it would increase costs and increase the difficulty of statistical analyses due to female cycles [67]. Yet, spurious conclusions about the presence or absence of sex/gender differences based on a dichotomous variable can affect research funding, research directions, and health care decisions. Currently, the Canadian Institutes of Health Research and previously also the United States' National Institutes of Health have made progress in dissociating sex and gender as separate variables, exhorting researchers to consider the effects of each on health and diseases, and providing educational resources [68, 69]. From a statistical viewpoint, if we are interested in sex and gender effects on health outcomes, we need to improve validity, reduce measurement error, avoid bias due to problematic cut-points, bolster power, and achieve representative sampling. There is no sensible argument for the continued use of dichotomised tickbox sex/gender in health research. We are combining the well-established literature on the problems of dichotomising continuous variables with the well-established literature on the nature of sex and gender to argue that the current approach has a substantial, but remediable, challenge to overcome while simultaneously providing an array of practical measurement and analytic approaches to act as a guide for researchers to move forward.

## Supplementary Information

Supplementary Material 1.

Supplementary Material 2.

Supplementary Material 3.

## Data Availability

All data generated or analysed during this study are included in this published article [and its supplementary information files].
