# Supplementary material for: Checking assumptions: advancing the analysis of sex and gender in health sciences
Source: Biol Sex Differ. 2026 Jan 2;17:19. doi: 10.1186/s13293-025-00803-7 (PMC12866590; doi:10.1186/s13293-025-00803-7)
Supplement: Supplementary file 1 — Supplementary Material 1. [file 13293_2025_803_MOESM1_ESM.pdf]

Supplements_210607

Eva Unternaehrer

14/06/2021

Prepare R Environment

```
# install.packages
#install_github("dbaranger/InteractionPoweR")

#Load packages
library(devtools)
library(InteractionPoweR)
library(parallel)
library(dplyr)
library(dbplyr)
library(correlation)
```

Calculate Increase in Sample Size when using binary instead of continuous moderator

For more details on this function, see:

David Baranger (2021). InteractionPoweR: Power analysis for interactions via simulation. R package version 0.1.0.3. https://github.com/dbaranger/InteractionPoweR (https://github.com/dbaranger/InteractionPoweR)

```
# correlation effect size
r <- 0.1

# power interaction with binary moderator
pwr_inter_bin <- power_interaction(
  n.iter = 100,            # number of simulations per unique combination of input pa
rameters
  alpha = 0.05,             # alpha, for the power analysis
  N = seq(100,1600,by=200), # sample size
    r.x1x2.y = r,           # interaction effect to test (correlation between x1*x2 a
nd y)
  r.x1.y = r,               # correlation between x1 and y
  r.x2.y = r,               # correlation between x2 and y
  r.x1.x2 = r,              # correlation between x1 and x2
  transform.x1 = "binary",  # x1 made binary
  adjust.correlations = F)  # do not adjust correlationsto see impact of artificial d
ichotomosiation
```

```
## [1] "Checking for errors in inputs..."
## [1] "Performing 800 simulations"
```

```
## Warning: executing %dopar% sequentially: no parallel backend registered
```

```
pwr_inter_bin
```

```
##      N  pwr     min.lwr    min.upr    max.lwr   max.upr
## 1  100 0.09 -0.42455121 0.17063108 0.15591271 0.4500451
## 2  300 0.32 -0.21055101 0.09403346 0.06840526 0.2959891
## 3  500 0.40 -0.14802371 0.08962995 0.03965690 0.2877091
## 4  700 0.48 -0.12225628 0.09431352 0.05647387 0.2412715
## 5  900 0.65 -0.09308270 0.09992974 0.08522728 0.2194741
## 6 1100 0.74 -0.07130901 0.05337723 0.06302568 0.2273938
## 7 1300 0.83 -0.05966416 0.06925281 0.07911027 0.2142555
## 8 1500 0.85 -0.06525535 0.08950651 0.07453957 0.2200053
```

```
power_estimate(pwr_inter_bin, power_target = .8, x = "N")
```

```
## [1] 1280.606
```

```
# power interaction with continuous moderator
pwr_inter_cont <- power_interaction(
  n.iter = 100,            # number of simulations per unique combination of input pa
rameters
  alpha = 0.05,             # alpha, for the power analysis
  N = seq(100,1600,by=200), # sample size
  r.x1x2.y = r,           # interaction effect to test (correlation between x1*x2 and
y)
  r.x1.y = r,             # correlation between x1 and y
  r.x2.y = r,             # correlation between x2 and y
  r.x1.x2 = r)            # correlation between x1 and x2
```

```
## [1] "Checking for errors in inputs..."
## [1] "Performing 800 simulations"
```

```
pwr_inter_cont
```

```
##      N  pwr     min.lwr    min.upr    max.lwr   max.upr
## 1  100 0.14 -0.32128690 0.06604055 0.10063834 0.5849296
## 2  300 0.44 -0.20053031 0.10341718 0.06668589 0.3221219
## 3  500 0.62 -0.12223246 0.11220876 0.09451221 0.2931572
## 4  700 0.70 -0.10904823 0.11418742 0.10703923 0.2675889
## 5  900 0.88 -0.08798622 0.10344154 0.09249660 0.2697472
## 6 1100 0.92 -0.04800624 0.07393808 0.10415649 0.2519631
## 7 1300 0.95 -0.06750262 0.08949528 0.10273940 0.2533365
## 8 1500 0.98 -0.05558806 0.07989870 0.10100380 0.2426672
```

```
power_estimate(pwr_inter_cont, power_target = .8, x = "N")
```

```
## [1] 826.3786
```

Kruskal-Wallis Test: One-way non-parametric ANOVA

The Kruskal Wallis Test provides you with an opportunity to test differences between multiple groups. Let's assume participants have had the opportunity to check different gender boxes beyond male-female.

```
# Create Mock-Data
genders <- c("man", "transgender man", "two-spirited", "transgender women", "women")
gender <- factor(sample(genders, 100, replace = TRUE, prob = c(0.3, 0.1, 0.1, 0.1,0.3
)), levels = genders, labels = genders)
table(gender)
```

```
## gender
##                man   transgender man      two-spirited transgender women 
##                 40                 8                10                 8 
##              women 
##                 34
```

```
testosterone1 <- rnorm(100, 0, 4)
testosterone <- rep(NA, 100)

testosterone[gender=="man"] <- 18 + testosterone1[gender=="man"]
testosterone[gender=="transgender man"] <- 15 + testosterone1[gender=="transgender ma
n"]
testosterone[gender=="two-spirited"] <- 10 + testosterone1[gender=="two-spirited"]
testosterone[gender=="transgender women"] <- 8 + testosterone1[gender=="transgender w
omen"]
testosterone[gender=="women"] <- 1 + testosterone1[gender=="women"]

# Visualize Data
plot(testosterone ~ gender)
```

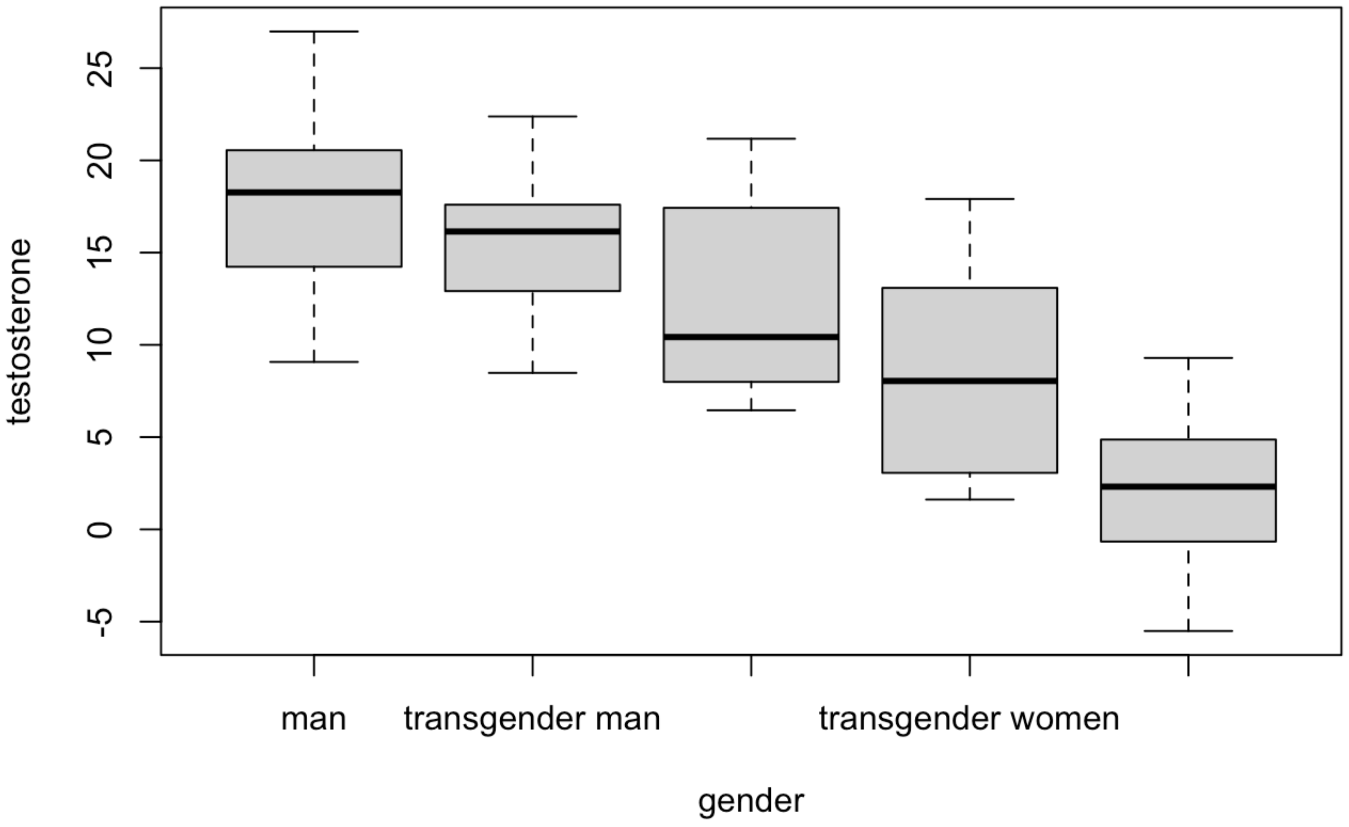

```r
# Omnibus Test
kruskal.test(testosterone ~ gender)
```

```
##
##  Kruskal-Wallis rank sum test
##
## data:  testosterone by gender
## Kruskal-Wallis chi-squared = 70.754, df = 4, p-value = 1.574e-14
```

```r
# Contrasts Test
pairwise.wilcox.test(testosterone,gender)
```

```
##
##  Pairwise comparisons using Wilcoxon rank sum exact test
##
## data:  testosterone and gender
##
##                   man     transgender man two-spirited transgender women
## transgender man   0.4233  -               -            -
## two-spirited      0.0178  0.4233          -            -
## transgender women 0.0011  0.1517          0.4233       -
## women             < 2e-16 3.1e-07         6.3e-07      0.0226
##
## P value adjustment method: holm
```

Correlation coefficients

We recommend using different forms of correlation coefficients when analyzig sex/gender, even when it was assessed dichotomously, because we assume an underlying continuous distribution. In particular, we recommend the following two approaches of correlation

- Biserial correlation: one variable is continuous and the sex/gender variable is dichotomous (but with assumed continuity)
- Polychoric correlation: correlation between two ordinal variables with assumed continuous normal distribution
- Tetrachoric correlation: both correlations were measured dichotomously.

For more details, see https://github.com/easystats/correlation (https://github.com/easystats/correlation).

```r
# Create Mock-Data
gender.bin <- sample(c(0,1), 100, replace = TRUE)

data <- data.frame(
  gender.bin=gender.bin,
  education=sample(c(0, 1), 100, replace = TRUE),
  size=170 + 5*gender.bin +rnorm(100, 0, 10))

# Biserial correlation (gender.bin is dichotomous, testosterone is continuous)
cor_test(data, "gender.bin", "size", method = "biserial")
```

```
## Parameter1 | Parameter2 |  rho |        95% CI | t(98) |     p
## ---------------------------------------------------------------
## gender.bin |       size | 0.13 | [-0.06, 0.32] |  1.35 | 0.182
##
## Observations: 100
```

```r
# Tetrachoric (gender.bin and education are both dichotomous)
cor_test(data, "gender.bin", "education", method = "tetrachoric")
```

```
## Parameter1 | Parameter2 |  rho |        95% CI | t(98) |     p
## ---------------------------------------------------------------
## gender.bin |  education | 0.03 | [-0.17, 0.23] |  0.31 | 0.755
##
## Observations: 100
```

```r
sessionInfo()
```

```
## R version 4.0.2 (2020-06-22)
## Platform: x86_64-apple-darwin17.0 (64-bit)
## Running under: macOS Catalina 10.15.5
##
## Matrix products: default
## BLAS:   /Library/Frameworks/R.framework/Versions/4.0/Resources/lib/libRblas.dylib
## LAPACK: /Library/Frameworks/R.framework/Versions/4.0/Resources/lib/libRlapack.dyli
b
##
## locale:
## [1] en_US.UTF-8/en_US.UTF-8/en_US.UTF-8/C/en_US.UTF-8/en_US.UTF-8
##
## attached base packages:
## [1] parallel  stats     graphics  grDevices utils     datasets  methods
## [8] base
##
## other attached packages:
## [1] MASS_7.3-51.6            correlation_0.6.1.1      dbplyr_1.4.4
```

```
## [4] dplyr_1.0.6             InteractionPoweR_0.1.0.3 devtools_2.4.0
## [7] usethis_2.0.1
##
## loaded via a namespace (and not attached):
##  [1] pkgload_1.2.1       splines_4.0.2       foreach_1.5.1
##  [4] tmvnsim_1.0-2       RhpcBLASctl_0.20-137 assertthat_0.2.1
##  [7] blob_1.2.1          kyotil_2021.5-14    yaml_2.2.1
## [10] remotes_2.4.0       bayestestR_0.10.5   sessioninfo_1.1.1
## [13] pillar_1.6.1        backports_1.1.8     lattice_0.20-41
## [16] glue_1.4.2          digest_0.6.27       minqa_1.2.4
## [19] sandwich_2.5-1      psych_2.1.3         htmltools_0.5.0
## [22] Matrix_1.2-18       pkgconfig_2.0.3     purrr_0.3.4
## [25] xtable_1.8-4        mvtnorm_1.1-1       processx_3.5.1
## [28] lme4_1.1-27         emmeans_1.4.8       chngpt_2021.5-12
## [31] tibble_3.1.2        generics_0.1.0      ellipsis_0.3.2
## [34] TH.data_1.0-10      cachem_1.0.4        withr_2.4.2
## [37] mnormt_2.0.1        cli_2.5.0           survival_3.1-12
## [40] magrittr_2.0.1      crayon_1.4.1        effectsize_0.4.5
## [43] memoise_2.0.0       estimability_1.3    evaluate_0.14
## [46] ps_1.6.0            fs_1.5.0            fansi_0.5.0
## [49] nlme_3.1-148        pkgbuild_1.2.0      tools_4.0.2
## [52] prettyunits_1.1.1   lifecycle_1.0.0     multcomp_1.4-13
## [55] stringr_1.4.0       callr_3.6.0         compiler_4.0.2
## [58] rlang_0.4.11        grid_4.0.2          nloptr_1.2.2.2
## [61] iterators_1.0.13    parameters_0.14.0.1 rmarkdown_2.3
## [64] boot_1.3-25         testthat_3.0.2      codetools_0.2-16
## [67] DBI_1.1.0           polynom_1.4-0       R6_2.5.0
## [70] zoo_1.8-8           knitr_1.29          fastmap_1.0.1
## [73] utf8_1.2.1          rprojroot_1.3-2     insight_0.14.1
## [76] desc_1.3.0          stringi_1.4.6       Rcpp_1.0.6
## [79] vctrs_0.3.8         tidyselect_1.1.1    xfun_0.15
## [82] coda_0.19-3
```
