# Supplementary material for: Checking assumptions: advancing the analysis of sex and gender in health sciences
Source: Biol Sex Differ. 2026 Jan 2;17:19. doi: 10.1186/s13293-025-00803-7 (PMC12866590; doi:10.1186/s13293-025-00803-7)
Supplement: Supplementary file 3 — Supplementary Material 3. [file 13293_2025_803_MOESM3_ESM.pdf]

Gender simulations real life examples

Eva Unternaehrer

08/04/2021

Overview

The following script illustrates how explained variance (R square) and power change if gender is analyzed as a dichotomized variable, given that the predictor of interest is in truth a continuous gender variable and that it is this continuous gender variable that is associated with the outcome, and not sex as a categorical variable, which is often used in research. In this script, we simulate data for evidence-based values of Cohen's D regarding differences between "sexes".

1. *varying difference in gender between male/man/masculine and female/woman/feminine:*

- CASE 1: d = 0.17 for masculine gender role based on assigned sex
- CASE 2: d = 0.27 for total serum T in 1-9 year olds based on assigned sex
- CASE 3: d = 0.48 for body esteem (weight) in 12-18 year olds based on “sex”
- CASE 4: d = 1.2 for total serum T in 10-11 year olds based on assigned sex
- CASE 5: d = 2.54 for plasma OT in mean age (35.1 +/- 7.5 for male; 34.9 +/- 6.2 for female)
- CASE 6: d = 2.7 for a composite of sex-typed play in childhood based on “early hormone environment”
- CASE 7: d = 3.7 for total serum T in 18-27 year olds based on assigned sex
- CASE 8: d = 11 for core gender identity based on “early hormone environment”

2. *varying strength of association between sex/gender and outcome:* going from no association (Outcome = 0 + **0*gender + error) to a strong negative or positive association (Outcome = 0 + |2|*gender** + error), with the error term being identical across simulations for comparability.

Simulate Data

Simulate Gender Data (independent variable)

```r
# Simulate Gender Data
## define function to set means of normal distribution at different locations

sim.gender <- function(x, Cohens=NULL){
  #Female/woman/feminine
  set.seed(135); f <- sample(x, 10000, replace=T)
  gender_f <- 0 + Cohens/2 +f
  data_f <- data.frame(gender=gender_f, sex=factor(rep("female", 10000),
                                                   levels=c("female", "male")))
  #Male/man/masculine
  set.seed(246); m <- sample(x, 10000, replace=T)
  gender_m <- m - (Cohens/2)
  #gender_m <- ifelse(gender_m>10, male_values-m, gender_m)
  data_m <- data.frame(gender=gender_m, sex=factor(rep("male", 10000),
                                                   levels=c("female", "male")))
  #Combine
  mydata <- rbind(data_f, data_m)
  return(mydata)
}

## Set parameters
### normal distributions to sample from (M=0, SD=1)
set.seed(111); r <- rnorm(100000, mean=0, sd=1)
hist(r)
```

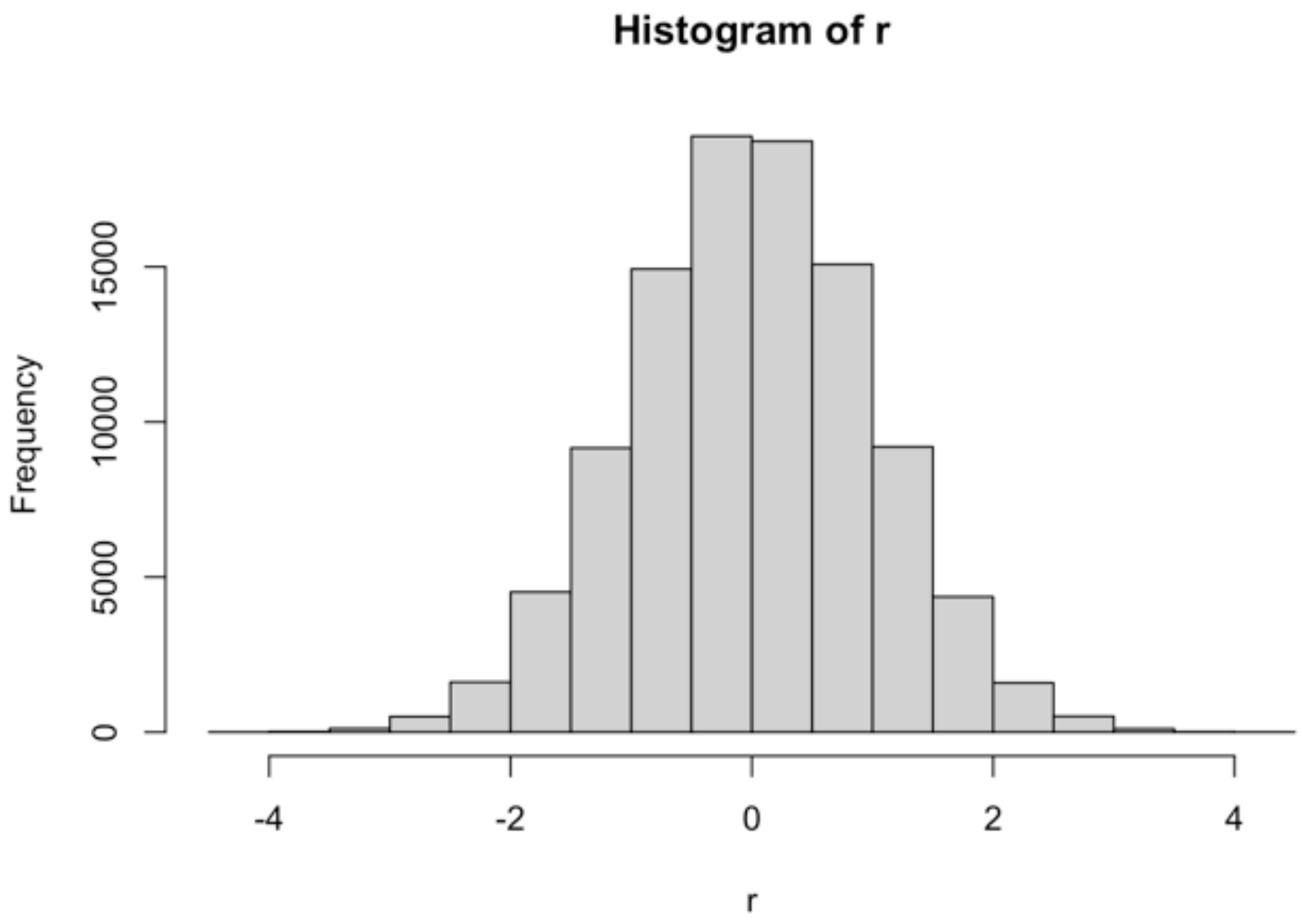

Histogram of r
Frequency
0
5000
10000
15000
-4
-2
0
2
4
r

```
diff <- c(11, 3.7, 2.7, 2.54, 1.2, 0.48, 0.27, 0.17)
colores <- c("red", "orange", "yellow", "green", "darkgreen", "turquoise", "blue", "p
urple")



### create empty vessel object
mylist_UV <- as.list(diff) # 8 CASES

## Run simulations
for (i in 1:length(diff)){
  mydata <- sim.gender(r, Cohens=diff[i])
  mylist_UV[[i]] <- mydata
}


###Plot distributions at various differences (8 CASES)
par(mfrow=c(3,3))
for (i in 1:length(diff)){
  mydata <- mylist_UV[[i]]
  mydata <- mydata[complete.cases(mydata),]
  plot_name <- paste ("F/W/F (straight) vs M/M/M (dashed) Distribution")
  sm.density.compare(mydata$gender, mydata$sex, xlab=plot_name, col=c(colores[i], col
ores[i]), lty=c(1,2), main=paste("Diff =", diff[i]))
}

#mydata <- mylist_UV[[2]]
#cohen.d(mydata$gender, group= mydata$sex)
```

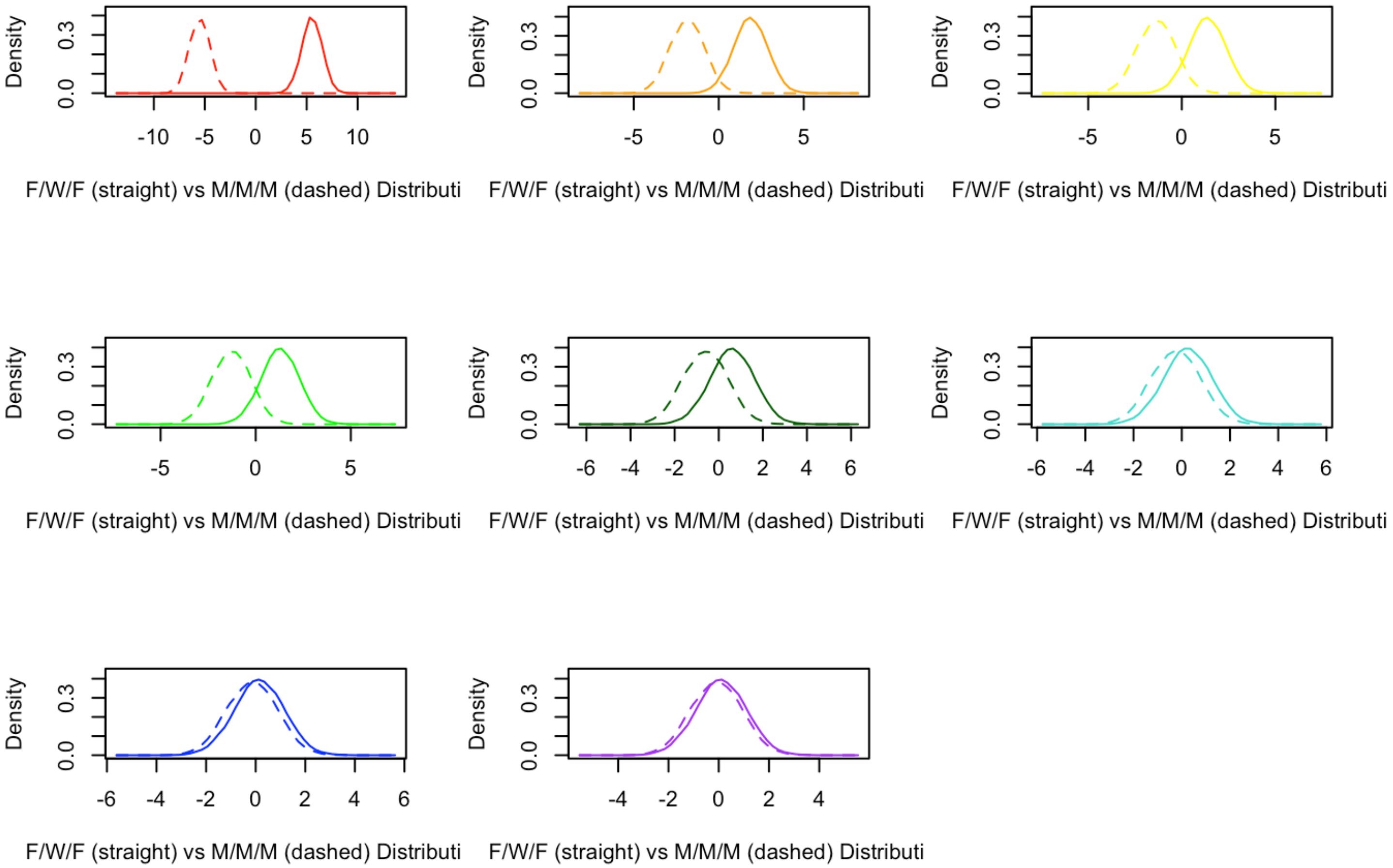

Missclassification Problem

The more overlap between the two distributions, the more miclassification we might see if a dichotmous predictor is used instead of a continuous predictor. If the two distributions do not overlap, then there is no danger of missclassification (CASE 1, and CASE 2). However, if the distributions are completely overlapping, then ~50% of the participants would be misclassified.

```
# Misclassification
tab_misclass <- data.frame(difference=factor(diff, levels=diff), misclassified = rep(
NA, times=length(diff)))

for (i in 1:length(diff)){
    mydata <- mylist_UV[[i]]
    mydata$classification <- as.factor(ifelse(mydata$gender<0, "classified male/man/m
asculine", "classified female/woman/feminine"))
    tab <- table(mydata$classification, mydata$sex)
    misclass <- prop.table(tab)[1,2] + prop.table(tab)[2,1]
    tab_misclass[i,2] <- misclass*100
}

b <- ggplot(tab_misclass, aes(x=difference, y=misclassified))
b + geom_bar(stat="identity", color=colores, fill=colores) +
  labs(title="", x="Mean Difference (Cohen's d)", y="Misclassified Individuals (%)")
+
  theme_bw() +
  theme(text = element_text(size = 18, color = "black"),
        axis.text.x = element_text(color = "black", size = 14),
        axis.text.y = element_text(color = "black", size = 14)) +
  scale_y_continuous(breaks = c(0, 10, 20, 30, 40, 50, 60), labels = c("0%", "10%", "
20%", "30%", "40%", "50%", "60%"), limits = c(0,50))
```

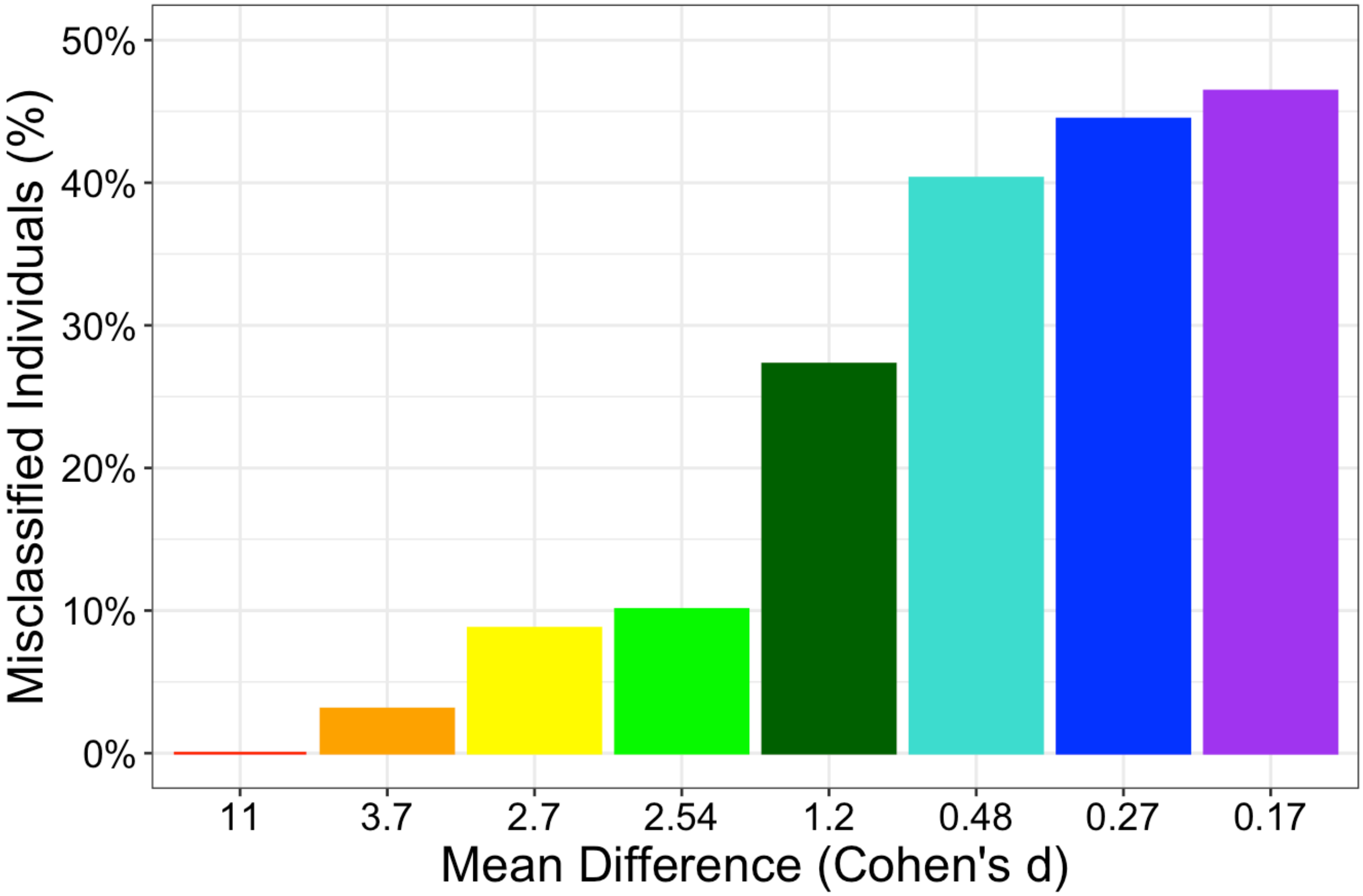

Simulate Outcome

The outcome data is simulated based on the assumption that there is a linear relationship between a continuous sex/gender variable and an outcome, with varying degrees of strength in the association between sex/gender and outcome, such that the smalles regression coefficient starts at 0 (no association) and ends with 2 (strong association) with scaled gender data (mean = 0, sd = 1).

```
# Simulate outcome data
## Define function to simulate outcome measures
sim.outcome <- function(df, effsize=NULL) {
  for (i in 1:length(effsize)){
      outcome <- as.numeric(scale(c(effsize[i] * scale(df$gender) + error)))
      name <- paste0("outcome_", effsize[i])
      df[2+i] <- outcome; names(df)[2+i] <- name
  }
  return(df)
}

## Set parameters
### define error term (constant for comparability)
set.seed(456); error <- rnorm(20000, mean=0, sd=1)
```

```
### define effect sizes
ES=seq(from=0, to=2, by = 0.05)

### create empty vessel objects
#gender
beta <- rep(NA, times=length(ES))
betas <- as.data.frame(matrix(ncol = length(diff), nrow = length(ES)))
names(betas) <- paste0("Difference_", diff)

## run sim.outcome function for all 8 CASES
mylist_DV <- as.list(1:length(diff))
for (i in 1:length(diff)){
  mydata <- sim.outcome(mylist_UV[[i]], ES)
  mylist_DV[[i]] <- mydata
}

mylist_deltabeta <- as.list(1:length(mylist_DV))
deltabeta <- rep(NA, length(ES))
mylist_deltabeta.p <- as.list(1:length(mylist_DV))
deltabeta.p <- rep(NA, length(ES))
mylist_R2 <- as.list(1:length(mylist_DV))
Rsquare <- rep(NA, length(ES))

for (j in 1:length(mylist_deltabeta)){
  # Select Dataframe
  mydata <- mylist_DV[[j]]

  # Models gender & sex
    for (i in 1:(ncol(mydata)-2)){
      #continuous sex/gender
      #gender <- mydata$gender
      #beta.g <- cor(as.matrix(cbind(gender, mydata[,i+2])),
      #               use="complete.obs")[1,2]
      beta.g <- cor(mydata$gender, as.numeric(mydata[,i+2]))
      beta.g.R2 <- beta.g*beta.g

      #dichotomous sex/gender
      #sex <- as.numeric(mydata$sex)
      beta.s <- cor(ifelse(mydata$sex=="female", 1, 0), mydata[,i+2])
      beta.s.R2 <- beta.s*beta.s

    deltabeta[i] <- beta.g-beta.s #difference
    deltabeta.p[i] <- deltabeta[i]/beta.g #difference(%)

    Rsquare[i] <- beta.g.R2 #R2
    }
  mylist_deltabeta.p[[j]] <- deltabeta.p
  mylist_deltabeta[[j]] <- deltabeta
  mylist_R2[[j]] <- Rsquare
  names(mylist_deltabeta)[j] <- paste0("Diff_", diff[j])
```

```
  names(mylist_deltabeta.p)[j] <- paste0("Diff_", diff[j])
  names(mylist_R2)[j] <- paste0("Diff_", diff[j])
}

mylist_deltabeta.df <- data.frame(mylist_deltabeta)
mylist_deltabeta.df$ES <- ES
#head(mylist_deltabeta.df)

mylist_deltabeta.p.df <- data.frame(mylist_deltabeta.p)
mylist_deltabeta.p.df$ES <- ES
#head(mylist_deltabeta.p.df)


mylist_R2.df <- data.frame(mylist_R2)
mylist_R2.df$ES <- ES
#head(mylist_R2.df)

###Graph deltabetas
g1 <- ggplot(data=mylist_deltabeta.df, aes(x=mylist_R2.df[,1], y=mylist_deltabeta.df[
,1], min=0, max=1, color=colores[1]))
g1 +
  geom_line() +
  geom_line(aes(x=mylist_R2.df[,2], y=mylist_deltabeta.df[,2], color=colores[2])) +
  geom_line(aes(x=mylist_R2.df[,3], y=mylist_deltabeta.df[,3], color=colores[3])) +
  geom_line(aes(x=mylist_R2.df[,4], y=mylist_deltabeta.df[,4], color=colores[4])) +
  geom_line(aes(x=mylist_R2.df[,5], y=mylist_deltabeta.df[,5], color=colores[5])) +
  geom_line(aes(x=mylist_R2.df[,6], y=mylist_deltabeta.df[,6], color=colores[6])) +
  geom_line(aes(x=mylist_R2.df[,7], y=mylist_deltabeta.df[,7], color=colores[7])) +
  geom_line(aes(x=mylist_R2.df[,8], y=mylist_deltabeta.df[,8], color=colores[8])) +
  labs(x="R squared", y="absolute difference in beta") +
  theme_bw() +
   theme(text = element_text(size = 18, color = "black"),
        axis.text.x = element_text(color = "black", size = 14),
        axis.text.y = element_text(color = "black", size = 14)) +
  scale_colour_manual(name = 'Difference',
                      values =c('red'='red','orange'='orange',
                                'yellow'='yellow','green'='green',
                                'darkgreen'='darkgreen',
                                'turquoise'='turquoise', 'blue'='blue',
                                'purple'='purple'),
                      breaks =c('red','orange', 'yellow', 'green',
                                'darkgreen', 'turquoise', 'blue', 'purple'),
                      labels=paste0("Difference = ", diff),
                      guide = "legend")
```

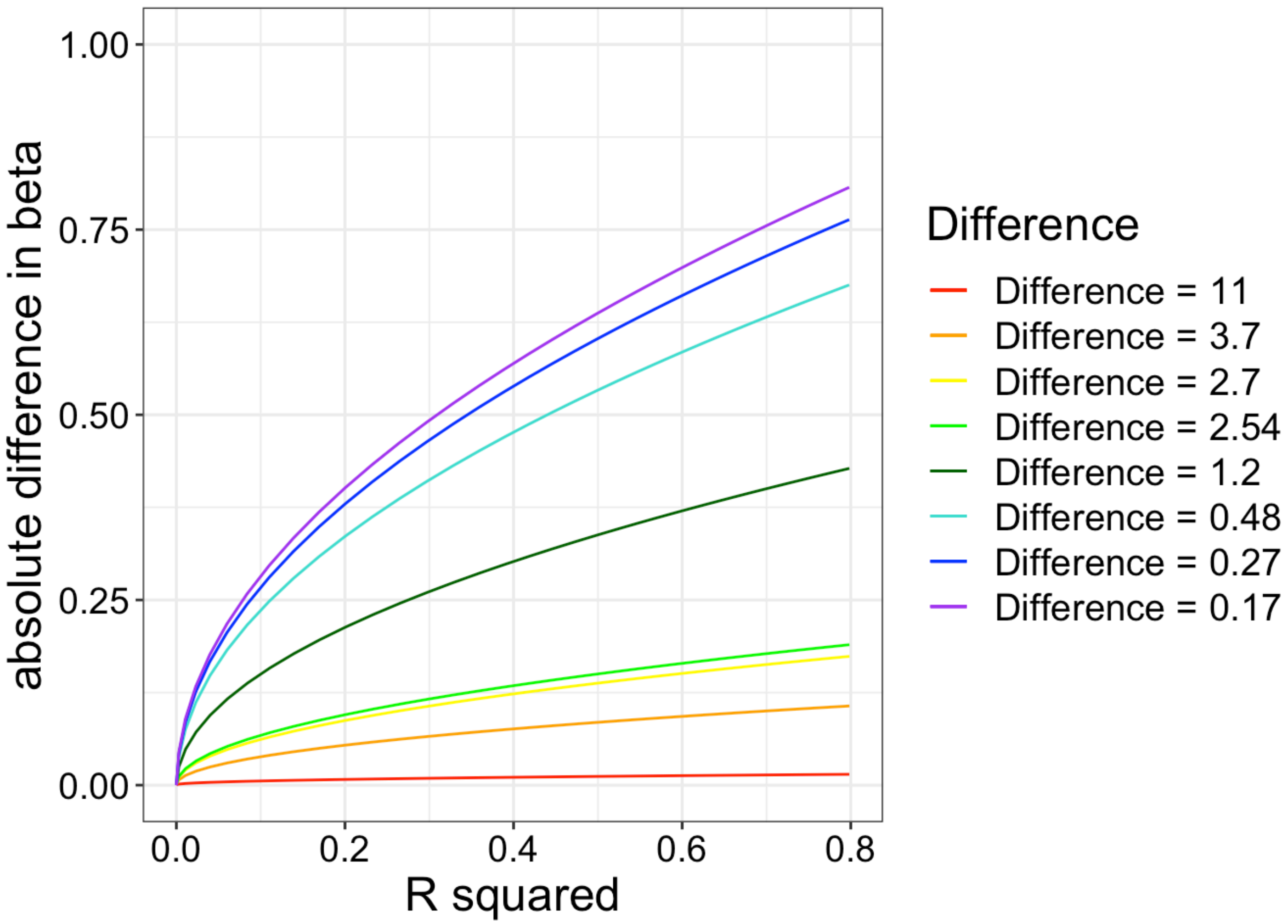

1.00
0.75
0.50
0.25
0.00
absolute difference in beta
0.0
0.2
0.4
0.6
0.8
R squared
Difference
Difference = 11
Difference = 3.7
Difference = 2.7
Difference = 2.54
Difference = 1.2
Difference = 0.48
Difference = 0.27
Difference = 0.17

```
g2 <- ggplot(data=mylist_deltabeta.p.df, aes(x=mylist_R2.df[,1], y=mylist_deltabeta.p
.df[,1], min=0, max=max(mylist_deltabeta.p.df[,1:length(diff)]), color=colores[1]))
g2 +
  geom_line() +
  geom_line(aes(x=mylist_R2.df[,2], y=mylist_deltabeta.p.df[,2], color=colores[2])) +
  geom_line(aes(x=mylist_R2.df[,3], y=mylist_deltabeta.p.df[,3], color=colores[3])) +
  geom_line(aes(x=mylist_R2.df[,4], y=mylist_deltabeta.p.df[,4], color=colores[4])) +
  geom_line(aes(x=mylist_R2.df[,5], y=mylist_deltabeta.p.df[,5], color=colores[5])) +
  geom_line(aes(x=mylist_R2.df[,6], y=mylist_deltabeta.p.df[,6], color=colores[6])) +
  geom_line(aes(x=mylist_R2.df[,7], y=mylist_deltabeta.p.df[,7], color=colores[7])) +
  geom_line(aes(x=mylist_R2.df[,8], y=mylist_deltabeta.p.df[,8], color=colores[8])) +
  labs(x="R squared", y="% difference in beta") +
  theme_bw() +
  xlim(0, 0.03) +
  theme(text = element_text(size = 18, color = "black"),
        axis.text.x = element_text(color = "black", size = 14),
        axis.text.y = element_text(color = "black", size = 14)) +
  scale_y_continuous(breaks = c(-0.15, 0, 0.25, 0.5, 0.75, 1.0), labels = c("", "0%",
"25%", "50%", "75%", "100%"), limits = c(-0.15,1))+
  scale_colour_manual(name = 'Difference',
                      values =c('red'='red','orange'='orange',
                                'yellow'='yellow','green'='green',
                                'darkgreen'='darkgreen',
                                'turquoise'='turquoise', 'blue'='blue',
                                'purple'='purple'),
                      breaks =c('red','orange', 'yellow', 'green',
                                'darkgreen', 'turquoise', 'blue', 'purple'),
                      labels=paste0("Difference = ", diff),
                      guide = "legend")
```

```
## Warning: Removed 37 row(s) containing missing values (geom_path).

## Warning: Removed 37 row(s) containing missing values (geom_path).

## Warning: Removed 37 row(s) containing missing values (geom_path).

## Warning: Removed 37 row(s) containing missing values (geom_path).

## Warning: Removed 37 row(s) containing missing values (geom_path).

## Warning: Removed 37 row(s) containing missing values (geom_path).

## Warning: Removed 37 row(s) containing missing values (geom_path).

## Warning: Removed 37 row(s) containing missing values (geom_path).
```

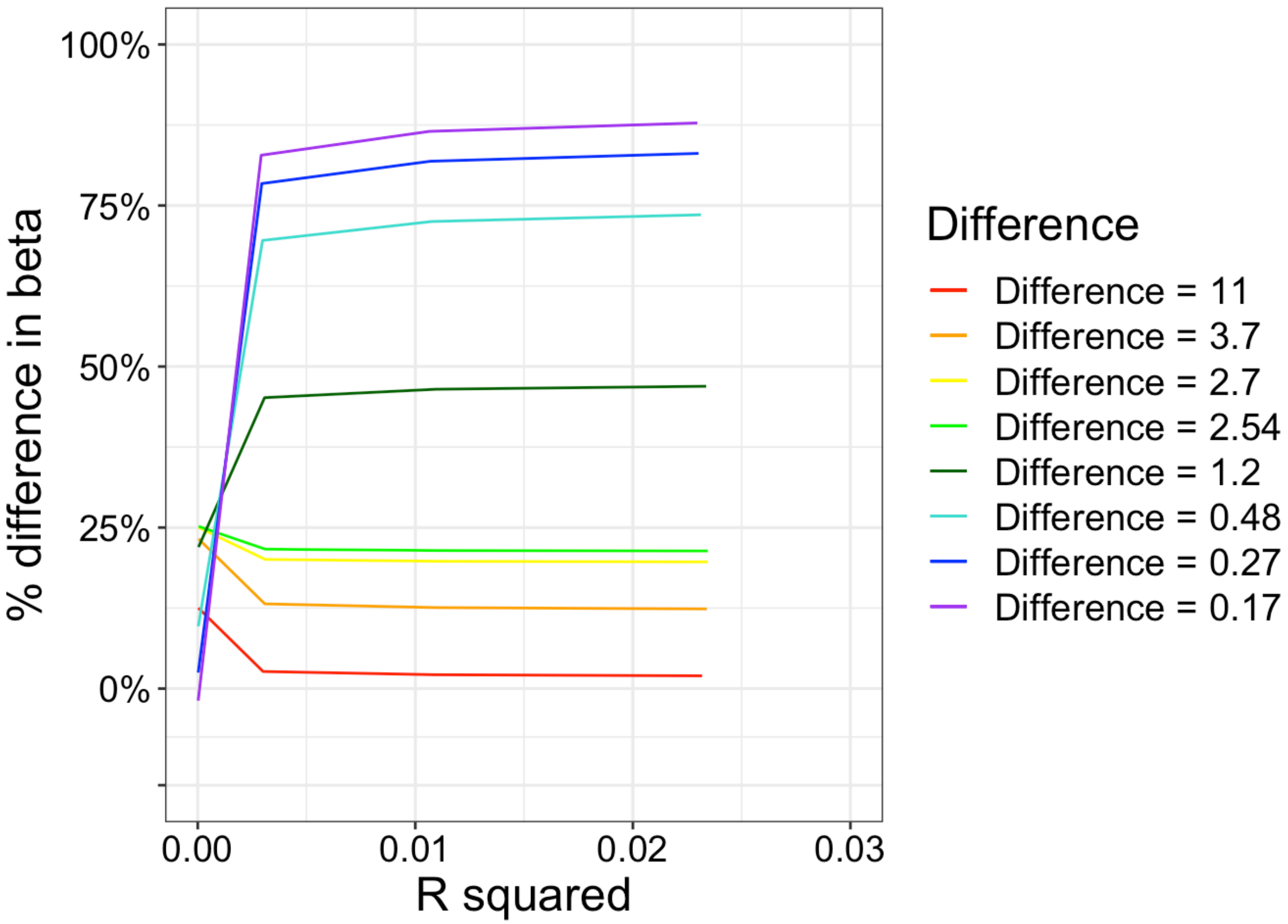

Compare Continous Sex/Gender vs. Dichotomous Sex/Gender Effect Sizes (explained variance, R2) and Power (PWR)

Calculation of Difference in R2 and Power between Dichotomous Sex/Gender and Continous Sex/Gender

The differences are calculated for R-squared and the Power estimates from the linear models, predicting the outcome by Dichotomous Sex/Gender minus predicting the outcome by Continous Sex/Gender:

$$delta = lm(outcome = sex + error) - lm(outcome = gender + error)$$

Random Sample Selection of N=100

The sample is drawn from the simulated data generated at various degrees of differences in the continuous sex/gender variable between dichotomous sex/gender variable. From this simulated data (let's assume the datasets of the 6 CASES are the population of interest), we draw random samples of n=100 to generate a random data sets. For each set, the identical samples are drawn to make the model outcomes (R square and Power) comparable. To obtain 95% confidence intervals, we repeated the sampling 1000 times.

```
# Random Data Sampling
## Set Parameters
### generate matrix with 1000 random samples (rows), each consisting of n=100 cases (
columns)
boots <- 1000 #number of bootstraps
sample.n <- 100 #number of bootstraps

indices <- matrix(nrow=boots, ncol=sample.n)
set.seed(123); seed_number <- sample(1:1000, boots, replace = F) #generate constant s
eeds with sample index from the n=20'000 file

for(i in 1:boots){
  set.seed(seed_number[i]); indices[i,] <- sample(1:nrow(mylist_DV[[1]]), sample.n, r
eplace = F)
}

#Comment: the seeds were pseudo-randomly generated, to obtain the same model paramete
rs if rerunning the script!

### Create empty vessel objects
difference <- rep(NA, times = boots)
diff_R2 <- difference; diff_R2_p <- difference; diff_PWR <- difference; diff_p <- dif
ference; diff_sig <- difference

p_vals_gender <- difference
p_vals_sex <- difference

ES_name <- paste("ES", ES, sep="_")
dfdiff <- as.data.frame(matrix(nrow = boots, ncol=length(ES))); names(dfdiff) <- ES_n
ame
dfdiff_R2 <- dfdiff; dfdiff_R2_p <- dfdiff; dfdiff_PWR <- dfdiff; dfdiff_p <- dfdiff;
dfdiff_sig <- dfdiff

dfpvals_g <- dfdiff
dfpvals_s <- dfdiff

listdfdiff <- as.list(paste0("Difference=", diff))
listdfdiff_R2 <- listdfdiff; listdfdiff_R2_p <- listdfdiff; listdfdiff_PWR <- listdfd
iff; listdfdiff_p <- listdfdiff; listdfdiff_sig <- listdfdiff

listdf_pvals_g <- listdfdiff
listdf_pvals_s <- listdfdiff

is.sig <- function(x){return(ifelse(x < 0.05, 1, 0))}
```

```
### draw 1000 bootstrapping samples * 41 effect sizes * 8 Differences
for(i in 1:length(diff)){
  mydata <- mylist_DV[[i]]
    for(j in 1:length(ES)){
      y <- names(mydata)[j+2]
        for(k in 1:boots){
          case_idx <- indices[k,] # select n=100
          d <- mydata[case_idx, ] # sample selection
          outcome <- d[,which(names(d)==y)]
          fit_g <- lm(outcome~d$gender)
          R2_g <- summary(fit_g)$r.squared
          f2_g <- R2_g/(1-R2_g)
          pwr_g <- pwr.f2.test(u=1, v=fit_g$df.residual, f2=f2_g, sig.level=0.05)$pow
er
          p_g <- summary(fit_g)$coefficients[2,4]
          sig_g <- is.sig(p_g)
          #Dichotomous Model
          fit_s <- lm(outcome~d$sex)
          R2_s <- summary(fit_s)$r.squared
          f2_s <- R2_s/(1-R2_s)
          pwr_s <- pwr.f2.test(u=1, v=fit_s$df.residual, f2=f2_s, sig.level=0.05)$pow
er
          p_s <- summary(fit_s)$coefficients[2,4]
          sig_s <- is.sig(p_s)
          #Differences
          diff_R2[k]<- R2_s - R2_g #Difference R square
          diff_R2_p[k]<- (R2_g-R2_s)/R2_s
          diff_PWR[k]<- pwr_s - pwr_g #Difference Power
          diff_p[k] <- p_s - p_g #for p-value Hackers
          diff_sig[k] <- sig_s - sig_g
          p_vals_gender[k] <- p_g
          p_vals_sex[k] <- p_s
        }
    dfdiff_R2[,j] <- diff_R2
    dfdiff_R2_p[,j] <- diff_R2_p
    dfdiff_PWR[,j] <- diff_PWR
    dfdiff_p[,j] <- diff_p
    dfdiff_sig[,j] <- diff_sig
    dfpvals_g[,j] <- p_vals_gender
    dfpvals_s[,j] <- p_vals_sex
    }
  listdfdiff_R2[[i]] <- dfdiff_R2
  listdfdiff_R2_p[[i]] <- dfdiff_R2_p
  listdfdiff_PWR[[i]] <- dfdiff_PWR
  listdfdiff_p[[i]] <-dfdiff_p
  listdfdiff_sig[[i]] <-dfdiff_sig
  listdf_pvals_g[[i]] <- dfpvals_g
  listdf_pvals_s[[i]] <- dfpvals_s
}
```

Plotting Differences in R square and Power

Here, the difference in R square and power are plotted as a function of effect size. More negative values mean that the loss in R square/Power is greater if dichotomous sex/gender is used instead of continuous sex/gender.

```
# Plotting Differences in R square and Power
## Define function to generate means, standard-deviations, standard errors and confid
ence intervals for plotting
### calculate betas
BETAS <- as.data.frame(matrix(ncol = length(diff), nrow = length(ES)))
names(BETAS) <- paste0("Diff_", diff)
rownames(BETAS) <- ES
beta <- rep(NA, length(ES))

# continous sex/gender
for(i in 1:length(diff)){
  mydata <- mylist_DV[[i]]
    for(j in 1:length(ES)){
      beta[j] <- coef(lm(mydata[,j+2]~ scale(mydata$gender) - 1))
    }
  BETAS[,i] <- round(beta, digits=4)
}


desc.fun <- function(data){
    ES <- seq(from=-2, to = 2, by=0.1)
    mean <- rowMeans(data)
    sd <- apply(data, 1, sd)
    error <- apply(data, 1, function(x) (qnorm(0.975)*sd(x))/sqrt(length(x)))
    ci_upper <- mean+error
    ci_lower <- mean-error
    desc <- data.frame(RegCoeff=BETAS[,2],
                       mean=mean, se=error,
                       ci_up=ci_upper, ci_lo=ci_lower)
    return(desc)
}

## Set Parameters
### Create empty vessel objects
list.desc <- as.list(1:length(diff))
list.desc_R2 <- list.desc; list.desc_R2_p <- list.desc; list.desc_PWR <- list.desc

## Calculate descriptive values for plotting
for(i in 1:length(diff)){
  list.desc_R2[[i]] <- desc.fun(t(listdfdiff_R2[[i]]))
  list.desc_R2_p[[i]] <- desc.fun(t(listdfdiff_R2_p[[i]]))
  list.desc_PWR[[i]] <- desc.fun(t(listdfdiff_PWR[[i]]))
}
```

```
#Graph for R square:
g <- ggplot(data=list.desc_R2[[1]], aes(x=RegCoeff, y=mean, min=-1, max=0, color=colo
res[1]))
g +
  geom_line(color=colores[1]) +
  geom_ribbon(data=list.desc_R2[[1]], aes(ymin=ci_lo,ymax=ci_up), alpha=0.5, color=co
lores[1], fill=colores[1]) +
  geom_line(data = list.desc_R2[[2]], aes(x=RegCoeff, y=mean), color=colores[2]) +
  geom_ribbon(data=list.desc_R2[[2]], aes(ymin=ci_lo,ymax=ci_up), alpha=0.5, color=co
lores[2], fill=colores[2]) +
  geom_line(data = list.desc_R2[[3]], aes(x=RegCoeff, y=mean), color=colores[3]) +
  geom_ribbon(data=list.desc_R2[[3]], aes(ymin=ci_lo,ymax=ci_up), alpha=0.5, color=co
lores[3], fill=colores[3]) +
  geom_line(data = list.desc_R2[[4]], aes(x=RegCoeff, y=mean), color=colores[4]) +
  geom_ribbon(data=list.desc_R2[[4]], aes(ymin=ci_lo,ymax=ci_up), alpha=0.5, color=co
lores[4], fill=colores[4]) +
  geom_line(data = list.desc_R2[[5]], aes(x=RegCoeff, y=mean), color=colores[5]) +
  geom_ribbon(data=list.desc_R2[[5]], aes(ymin=ci_lo,ymax=ci_up), alpha=0.5, color=co
lores[5], fill=colores[5]) +
  geom_line(data = list.desc_R2[[6]], aes(x=RegCoeff, y=mean), color=colores[6]) +
  geom_ribbon(data=list.desc_R2[[6]], aes(ymin=ci_lo,ymax=ci_up), alpha=0.5, color=co
lores[6], fill=colores[6]) +
  geom_line(data = list.desc_R2[[7]], aes(x=RegCoeff, y=mean), color=colores[7]) +
  geom_ribbon(data=list.desc_R2[[7]], aes(ymin=ci_lo,ymax=ci_up), alpha=0.5, color=co
lores[7], fill=colores[7]) +
  geom_line(data = list.desc_R2[[8]], aes(x=RegCoeff, y=mean), color=colores[8]) +
  geom_ribbon(data=list.desc_R2[[8]], aes(ymin=ci_lo,ymax=ci_up), alpha=0.5, color=co
lores[8], fill=colores[8]) +
  scale_colour_manual(name = 'Difference',
                     values =c('red'='red','orange'='orange',
                                'yellow'='yellow','green'='green',
                                'darkgreen'='darkgreen',
                                'turquoise'='turquoise', 'blue'='blue',
                                'purple'='purple'),
                      breaks =c('red','orange', 'yellow', 'green',
                                'darkgreen', 'turquoise', 'blue', 'purple'),
                      labels=paste0("Difference = ", diff),
                      guide = "legend") +
  theme_bw() +
  labs(x = "Standardized Regression Coefficients", y="Mean Loss in Rsquared")+
  theme(text = element_text(size = 18, color = "black"),
        axis.text.x = element_text(color = "black", size = 14),
        axis.text.y = element_text(color = "black", size = 14))
```

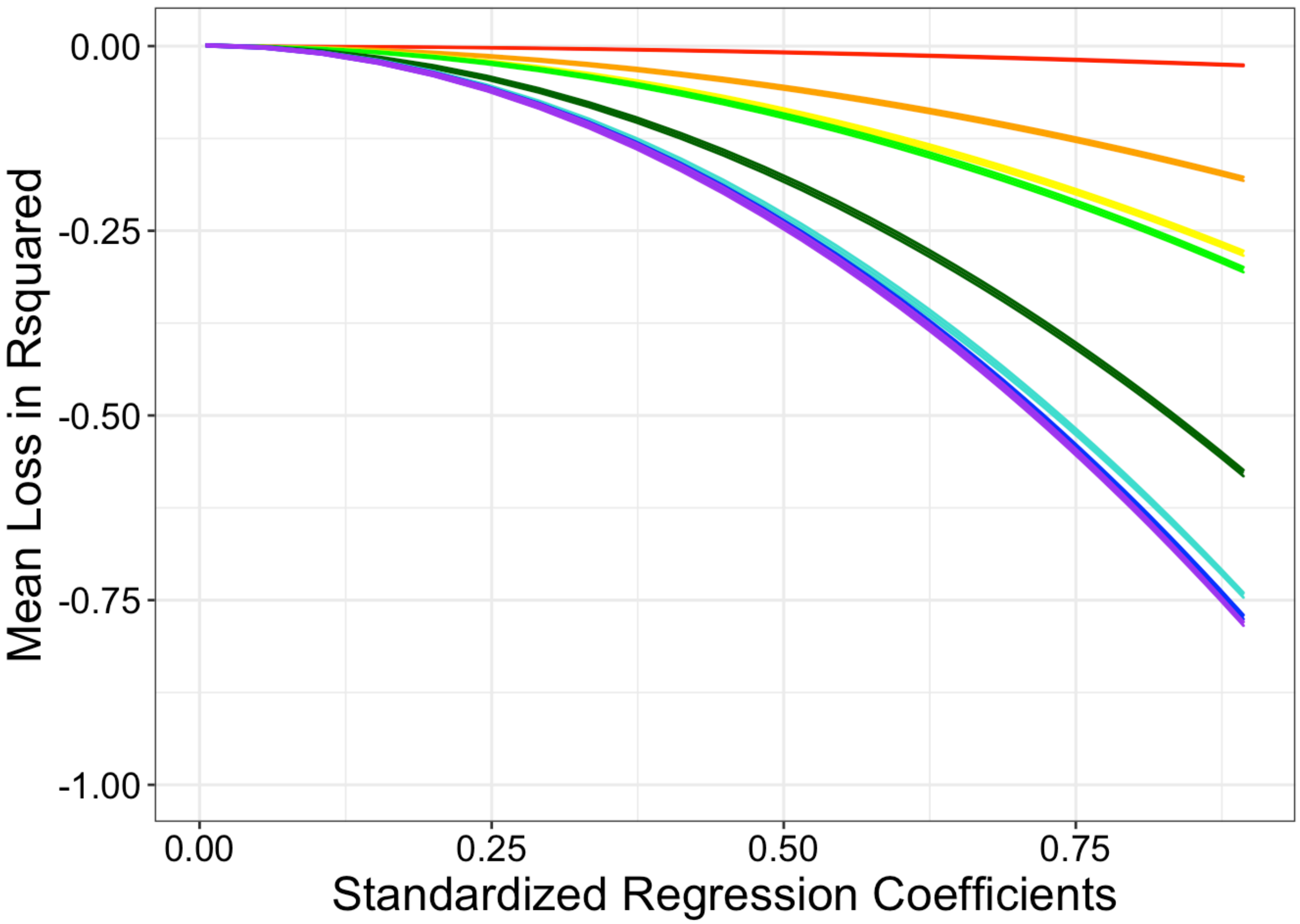

0.00
-0.25
-0.50
-0.75
-1.00
Mean Loss in Rsquared
0.00
0.25
0.50
0.75
Standardized Regression Coefficients

```
#Graph for PWR:
g <- ggplot(data=list.desc_PWR[[1]], aes(x=RegCoeff, y=mean, min=-1, max=0, colour =
colores[1]))
g +
  geom_line(color=colores[1]) +
  geom_ribbon(data=list.desc_PWR[[1]], aes(ymin=ci_lo,ymax=ci_up), alpha=0.5, color=c
olores[1], fill=colores[1]) +
  geom_line(data = list.desc_PWR[[2]], aes(x=RegCoeff, y=mean), color=colores[2]) +
  geom_ribbon(data=list.desc_PWR[[2]], aes(ymin=ci_lo,ymax=ci_up), alpha=0.5, color=c
olores[2], fill=colores[2]) +
  geom_line(data = list.desc_PWR[[3]], aes(x=RegCoeff, y=mean), color=colores[3]) +
  geom_ribbon(data=list.desc_PWR[[3]], aes(ymin=ci_lo,ymax=ci_up), alpha=0.5, color=c
olores[3], fill=colores[3]) +
  geom_line(data = list.desc_PWR[[4]], aes(x=RegCoeff, y=mean), color=colores[4]) +
  geom_ribbon(data=list.desc_PWR[[4]], aes(ymin=ci_lo,ymax=ci_up), alpha=0.5, color=c
olores[4], fill=colores[4]) +
  geom_line(data = list.desc_PWR[[5]], aes(x=RegCoeff, y=mean), color=colores[5]) +
  geom_ribbon(data=list.desc_PWR[[5]], aes(ymin=ci_lo,ymax=ci_up), alpha=0.5, color=c
olores[5], fill=colores[5]) +
  geom_line(data = list.desc_PWR[[6]], aes(x=RegCoeff, y=mean), color=colores[6]) +
  geom_ribbon(data=list.desc_PWR[[6]], aes(ymin=ci_lo,ymax=ci_up), alpha=0.5, color=c
olores[6], fill=colores[6]) +
  geom_line(data = list.desc_PWR[[7]], aes(x=RegCoeff, y=mean), color=colores[7]) +
  geom_ribbon(data=list.desc_PWR[[7]], aes(ymin=ci_lo,ymax=ci_up), alpha=0.5, color=c
olores[7], fill=colores[7]) +
  geom_line(data = list.desc_PWR[[8]], aes(x=RegCoeff, y=mean), color=colores[8]) +
  geom_ribbon(data=list.desc_PWR[[8]], aes(ymin=ci_lo,ymax=ci_up), alpha=0.5, color=c
olores[8], fill=colores[8]) +
  theme_bw() +
  labs(x = "Standardized Regression Coefficients", y="Mean Loss in Power") +
  theme(text = element_text(size = 18, color = "black"),
        axis.text.x = element_text(color = "black", size = 14),
        axis.text.y = element_text(color = "black", size = 14)) +
  scale_colour_manual(name = 'Difference',
                      values =c('red'='red','orange'='orange',
                                'yellow'='yellow','green'='green',
                                'darkgreen'='darkgreen',
                                'turquoise'='turquoise', 'blue'='blue',
                                'purple'='purple'),
                      breaks =c('red','orange', 'yellow', 'green',
                                'darkgreen', 'turquoise', 'blue', 'purple'),
                      labels=paste0("Difference = ", diff),
                      guide = "legend")
```

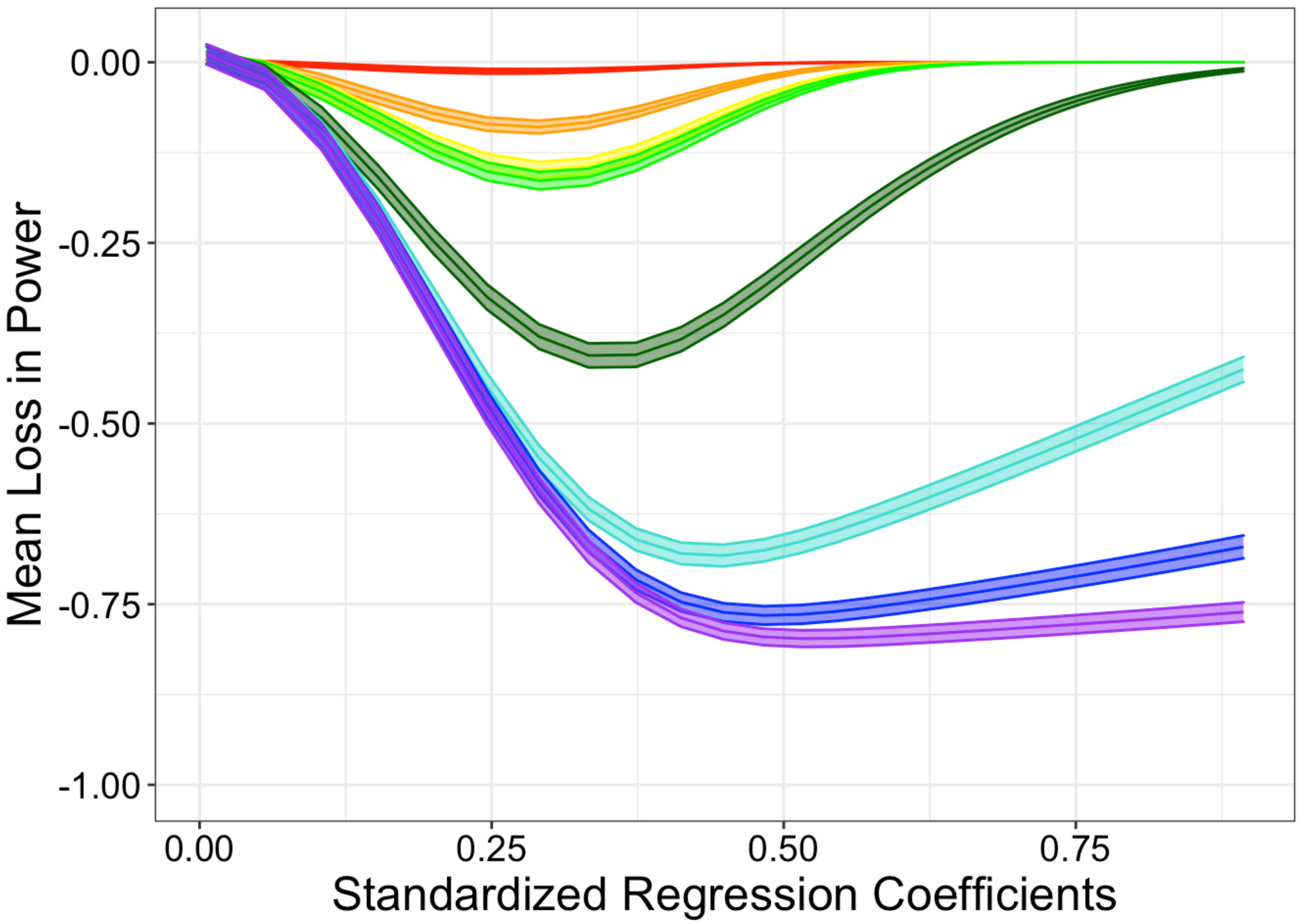

Session Info

```
sessionInfo()
```

```
## R version 4.0.2 (2020-06-22)
## Platform: x86_64-apple-darwin17.0 (64-bit)
## Running under: macOS Catalina 10.15.5
##
## Matrix products: default
## BLAS:   /Library/Frameworks/R.framework/Versions/4.0/Resources/lib/libRblas.dylib
## LAPACK: /Library/Frameworks/R.framework/Versions/4.0/Resources/lib/libRlapack.dyli
b
##
## locale:
## [1] en_US.UTF-8/en_US.UTF-8/en_US.UTF-8/C/en_US.UTF-8/en_US.UTF-8
##
## attached base packages:
## [1] stats     graphics  grDevices utils     datasets  methods   base
##
## other attached packages:
## [1] sm_2.2-5.6    QuantPsyc_1.5 MASS_7.3-51.6 boot_1.3-25   ggplot2_3.3.2
## [6] pwr_1.3-0
##
## loaded via a namespace (and not attached):
##  [1] pillar_1.4.4     compiler_4.0.2   tools_4.0.2      digest_0.6.25
##  [5] evaluate_0.14    lifecycle_1.0.0  tibble_3.0.2     gtable_0.3.0
##  [9] pkgconfig_2.0.3  rlang_0.4.10     yaml_2.2.1       xfun_0.15
## [13] withr_2.4.1      stringr_1.4.0    dplyr_1.0.0      knitr_1.29
## [17] generics_0.0.2   vctrs_0.3.1      grid_4.0.2       tidyselect_1.1.0
## [21] glue_1.4.1       R6_2.4.1         tcltk_4.0.2      rmarkdown_2.3
## [25] purrr_0.3.4      farver_2.0.3     magrittr_1.5     scales_1.1.1
## [29] ellipsis_0.3.1   htmltools_0.5.0  colorspace_1.4-1 labeling_0.3
## [33] stringi_1.4.6    munsell_0.5.0    crayon_1.3.4
```
